# Pericytes augment glioblastoma cell resistance to temozolomide through CCL5-CCR5 paracrine signaling

**DOI:** 10.1038/s41422-021-00528-3

**Published:** 2021-07-08

**Authors:** Xiao-Ning Zhang, Kai-Di Yang, Cong Chen, Zhi-Cheng He, Qiang-Hu Wang, Hua Feng, Sheng-Qing Lv, Yan Wang, Min Mao, Qing Liu, Yao-Yao Tan, Wen-Ying Wang, Tian-Ran Li, Lin-Rong Che, Zhong-Yi Qin, Ling-Xiang Wu, Min Luo, Chun-Hua Luo, Yu-Qi Liu, Wen Yin, Chao Wang, Hai-Tao Guo, Qing-Rui Li, Bin Wang, Wei Chen, Shuang Wang, Yu Shi, Xiu-Wu Bian, Yi-Fang Ping

**Affiliations:** 1 Institute of Pathology and Southwest Cancer Center, Southwest Hospital, Third Military Medical University (Army Medical University) and Key Laboratory of Tumor Immunopathology, Ministry of Education of China, Chongqing, China; 2 Department of Bioinformatics, Nanjing Medical University, Nanjing, Jiangsu China; 3 Department of Neurosurgery, Southwest Hospital, Third Military Medical University (Army Medical University), Chongqing, China; 4 Department of Neurosurgery, Xinqiao Hospital, Third Military Medical University (Army Medical University), Chongqing, China; 5 Department of Gastroenterology, Daping Hospital, Third Military Medical University (Army Medical University), Chongqing, China; 6 Department of Radiology, Southwest Hospital, Third Military Medical University (Army Medical University), Chongqing, China; 7 Department of Radiology, Xinqiao Hospital, Third Military Medical University (Army Medical University), Chongqing, China

**Keywords:** Cancer microenvironment, CNS cancer, Cancer therapy

## Abstract

Glioblastoma (GBM) is a prevalent and highly lethal form of glioma, with rapid tumor progression and frequent recurrence. Excessive outgrowth of pericytes in GBM governs the ecology of the perivascular niche, but their function in mediating chemoresistance has not been fully explored. Herein, we uncovered that pericytes potentiate DNA damage repair (DDR) in GBM cells residing in the perivascular niche, which induces temozolomide (TMZ) chemoresistance. We found that increased pericyte proportion correlates with accelerated tumor recurrence and worse prognosis. Genetic depletion of pericytes in GBM xenografts enhances TMZ-induced cytotoxicity and prolongs survival of tumor-bearing mice. Mechanistically, C-C motif chemokine ligand 5 (CCL5) secreted by pericytes activates C-C motif chemokine receptor 5 (CCR5) on GBM cells to enable DNA-dependent protein kinase catalytic subunit (DNA-PKcs)-mediated DDR upon TMZ treatment. Disrupting CCL5-CCR5 paracrine signaling through the brain-penetrable CCR5 antagonist maraviroc (MVC) potently inhibits pericyte-promoted DDR and effectively improves the chemotherapeutic efficacy of TMZ. GBM patient-derived xenografts with high CCL5 expression benefit from combined treatment with TMZ and MVC. Our study reveals the role of pericytes as an extrinsic stimulator potentiating DDR signaling in GBM cells and suggests that targeting CCL5-CCR5 signaling could be an effective therapeutic strategy to improve chemotherapeutic efficacy against GBM.

## Introduction

Glioblastoma (GBM) is the most prevalent and invariably fatal brain tumor.^1^ Abundant vasculature is a key characteristic of GBM and provides essential environmental cues to support tumor propagation and progression.^2^ Pericytes are auxiliary cells that wrap around the endothelial tubing of vessels and are critical elements of GBM vasculature. It has been well demonstrated that pericytes reciprocally interact with endothelial cells to regulate vascular functions under physiological contexts.^3^ Irregular pericytes are frequently observed in the dysfunctional vasculature of tumors. Emerging studies reveal that pericytes control tumor vascular stability and permeability.^4,5^ Pericyte coverage is associated with transmigration and infiltration of immune cells and intravasation of cancer cells thus affecting immune surveillance and tumor metastasis.^6–8^ Recent studies by us and others indicate that hyperplasia of pericytes contributes to vascular abnormity, which impacts anti-tumor drug delivery.^9^ Targeting glioma stem cell (GSC)-derived pericytes disrupts the blood–tumor barrier (BTB) in GBMs, resulting in increased effusion of chemotherapeutic agents.^10^ These results underscore the critical role of pericytes in maintaining the ecology of the tumor perivascular niche where both pericytes and tumor cells reside. However, whether pericytes directly interact with tumor cells to potentiate GBM malignant progression remains elusive.

GBM cells are highly resistant to conventional therapeutics including chemotherapy, which inevitably leads to tumor recurrence and poor prognosis. Temozolomide (TMZ), the first-line chemoagent for GBM treatment, efficiently penetrates the BTB and causes cytotoxicity through inducing DNA double-strand breaks.^11^ A major mechanism underlying the chemotherapeutic resistance of tumor cells is the canonical DNA damage repair response (DDR), initiated by activation of the kinases Ataxia-telangiectasia mutated (ATM), Ataxia-telangiectasia and Rad3-related (ATR) and DNA-dependent protein kinase catalytic subunit (DNA-PKcs).^12,13^ Identification of tumor-specific pathways underlying DDR initiation and hyperactivation is critical for the development of novel molecular targeting therapeutics. Increasing evidence implies that enhanced DDR in GBM cells is acquired not only through the alterations in endogenous gene expression patterns in tumors, but also through exogenous signals from the tumor microenvironment. In particular, the perivascular niche in GBMs provides environmental cues to fuel tumor growth, angiogenesis and invasion.^14^ As an important element of the perivascular niche, pericyte density is inversely correlated with the prognosis of patients treated with chemotherapy.^10^ However, whether pericytes communicate with GBM cells to promote chemoresistance has not been defined.

By interrogating the role of pericytes in regulating the therapeutic efficacy of TMZ, we found that disruption of pericytes had negligible impact on the delivery and penetration of TMZ into GBM xenografts. However, through screening soluble factors preferentially expressed by pericytes isolated from human GBMs, we found that C-C motif chemokine ligand 5 (CCL5) was abundantly secreted by GBM pericytes. CCL5 belongs to a chemokine superfamily and plays a pivotal role in regulating various physiological processes.^15^ Overexpression of CCL5 in multiple tumor types is associated with worse patient outcomes.^16,17^ CCL5 primarily functions through stimulating its receptor, C-C motif chemokine receptor 5 (CCR5), to trigger the activation of downstream RAC-alpha serine/threonine-protein kinase (AKT) and signal transducer and activator of transcription 3 (STAT3) pathways, thus promoting tumor malignant behaviors.^18^ However, the distribution of CCL5 in GBMs and the function of the CCL5-CCR5 signaling in regulating chemoresistance in GBM remains elusive.

In this study, we investigated the role of CCL5-CCR5 paracrine signaling in mediating the effects of pericytes on GBM chemoresistance. Furthermore, we evaluated the therapeutic potential of targeting CCL5-CCR5 signaling by employing the specific CCR5 antagonist maraviroc (MVC) for GBM treatment. MVC is a Food and Drug Administration (FDA)-approved agent that has been used clinically for the treatment of acquired immunodeficiency syndrome (AIDS).^19^ Importantly, MVC effectively penetrates the blood–brain barrier (BBB).^20^ Our preclinical data indicate that repurposing MVC for targeting CCL5-CCR5 signaling effectively improves the chemotherapeutic efficacy of TMZ against GBMs.

## Results

### Pericyte enrichment informs poor therapeutic response to TMZ in human GBMs

**Fig. 1:**
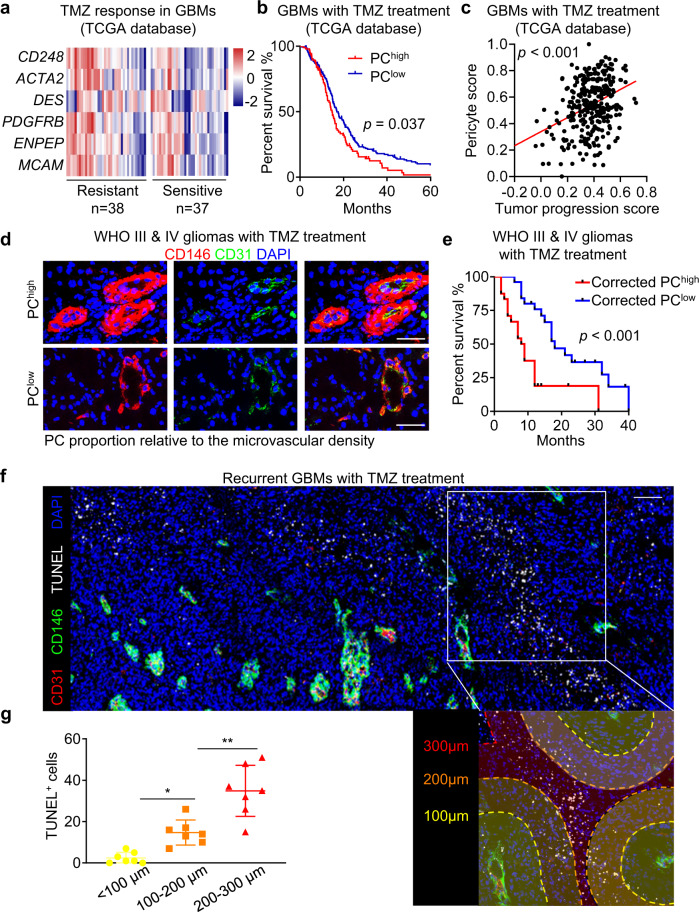
Enriched pericyte signature informs poor therapeutic efficacy of TMZ in glioma patients. **a** Heatmap of pericyte markers (*CD248*, *ACTA2*, *DES*, *PDGFRB*, *ENPEP* and *MCAM*) in TMZ-resistant (*n* = 38) and TMZ-sensitive GBMs (*n* = 37) from the TCGA database. **b** Kaplan–Meier survival analysis of the OS of TMZ-treated GBM patients stratified by pericyte score. Pericyte score was calculated according to the expression of pericyte (PC) markers in GBMs from the TCGA database (PC^high^, *n* = 96; PC^low^, *n* = 215). **c** Correlation analysis of pericyte score and tumor progression score in TMZ-treated GBMs from the TCGA database (*n* = 311). Tumor progression score was constructed by single sample gene set enrichment analysis (ssGSEA) model based on 14 signature genes derived from multi-focal GBM RNA-Seq. **d** Immunofluorescence staining of pericyte (PC) marker CD146 (red) and endothelial cell marker CD31 (green) in WHO III and IV gliomas treated with TMZ from Southwest Hospital. Scale bars, 50 μm. **e** Kaplan–Meier analysis of progression-free survival (PFS) of TMZ-treated WHO III and IV glioma patients stratified by pericyte level from Southwest Hospital. The corrected level of pericyte (PC) was defined as CD146-positive area/vessel number (PC^high^, *n* = 25; PC^low^, *n* = 24). **f** Immunofluorescence staining of pericyte marker CD146 (green) and endothelial cell marker CD31 (red), and TUNEL staining of apoptotic cells (white) in the recurrent GBMs treated with TMZ. DAPI, 4,6-diamidino-2-phenylindole; TUNEL, TdT-mediated dUTP nick-end labeling. Scale bars, 100 μm. **g** Quantification of TUNEL-positive cells at a distance of 100 μm, 200 μm or 300 μm from pericytes in the recurrent GBMs treated with TMZ. *P < 0.05; **P < 0.01.

Pericytes have been implicated as a potentiating factor in sustaining a favorable niche for tumor survival.^2,21^ To determine whether a pericyte signature in GBMs might be associated with the therapeutic efficacy of TMZ, we investigated The Cancer Genome Atlas (TCGA) database for GBMs treated with TMZ and stratified the cohort into TMZ-resistant GBMs (median overall survival (OS) < 14.2 months) and TMZ-sensitive GBMs (median OS > 14.2 months). We found an upregulation of expression of pericyte markers in TMZ-resistant GBMs relative to TMZ-sensitive GBMs (Fig. 1a). Higher pericyte proportion was associated with a worse outcome of patients with TMZ treatment, but not those without TMZ treatment (Fig. 1b; Supplementary information, Fig. S1a–c). In silico analysis indicated that pericyte enrichment was also associated with advanced tumor progression (Fig. 1c; Supplementary information, Fig. S1d). These data indicate that pericyte enrichment informs poor response to TMZ in human GBMs. To determine whether increased pericyte proportion is subsequent to high microvascular density, thus correlating with the reduced TMZ efficacy in GBMs, we performed immunofluorescence staining of the pericyte marker CD146^22^ and endothelial cell marker CD31 in malignant gliomas with TMZ treatment (Fig. 1d). The corrected proportion of pericytes in human GBMs was calculated as the ratio of CD146-positive pericyte density versus the CD31-positive microvascular density in the same area. The results demonstrated that pericyte enrichment and poor response to TMZ was independent of GBM microvascular density (Fig. 1e; Supplementary information, Fig. S1e). To determine whether the pericyte signature correlates with TMZ-induced cytotoxicity, we evaluated the apoptotic cell proportion and the proximity to pericytes in human recurrent GBMs treated with TMZ. Reduced cell apoptosis, marked by TdT-mediated dUTP nick-end labeling (TUNEL) staining, was observed in tumor areas proximal to pericytes, whereas an increase in apoptosis was observed in tumor areas distal to pericytes (Fig. 1f, g). Taken together, these results indicate that enriched pericytes inform poor therapeutic efficacy of TMZ in GBMs.

### Depletion of pericytes in pericyte-enriched GBM xenografts improves TMZ therapeutic efficacy but has negligible impact on TMZ penetration

**Fig. 2:**
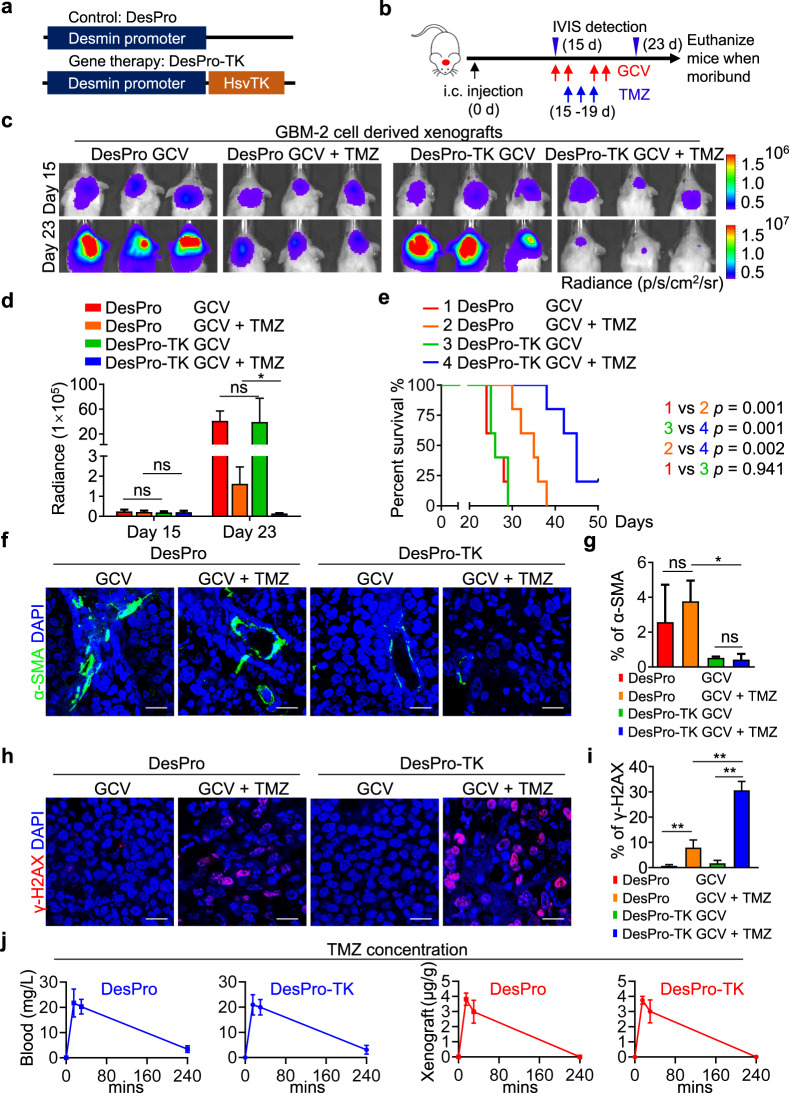
Depletion of pericytes in pericyte^high^ GBMs improves the therapeutic efficacy of TMZ. **a** Schematic diagram of Desmin promoter-driven expression of HsvTK vector and control vector. Gene therapy was achieved through administration of GCV, which is converted to a toxic metabolite to eliminate cells expressing HsvTK. **b** Schematic diagram of the combined treatment of TMZ and GCV in mice bearing GBM-2 (pericyte^high^) xenografts expressing DesPro-TK or control DesPro. GCV (50 mg/kg, i.p.) was given for 4 days with 1-day interval since Day 15 and TMZ (5 mg/kg, i.p.) was given for 3 consecutive days since Day 16 after tumor implantation. Xenograft growth was monitored by bioluminescence imaging on Day 15 and Day 23 after tumor implantation. IVIS, In Vivo Imaging System. **c**, **d** In vivo bioluminescence images (**c**) and quantification (**d**) of tumor growth of human GBM-2 xenografts treated with GCV together with or without TMZ on Day 15 and Day 23 after tumor implantation. ns, not significant. *P < 0.05. *n* = 5 for each group. **e** Kaplan–Meier survival analysis of mice bearing GBM-2 xenografts with the indicated treatment. *n* = 5 for each group. **f**, **g** Immunofluorescence staining (**f**) and quantification (**g**) of pericyte marker α-SMA (green) in GBM-2 xenografts treated with GCV together with or without TMZ. ns, not significant. *P < 0.05. Scale bars, 25 μm. **h**, **i** Immunofluorescence staining (**h**) and quantification (**i**) of γ-H2AX (red)-positive cells in GBM-2 xenografts treated with GCV together with or without TMZ. **P < 0.01. Scale bars, 25 μm. **j** TMZ concentration in GBM-2 xenografts and blood in mice expressing DesPro-TK or control DesPro with GCV treatment. *n* = 4 for each group.

Given that pericyte enrichment informs poor therapeutic efficacy of TMZ, we tested whether depletion of pericytes in GBM xenografts could improve TMZ efficacy. As the majority of pericytes in GBMs are derived from GSCs,^23^ we constructed GBM xenografts using GSCs (GBM-2) transduced with a Desmin promoter-driven herpes simplex virus thymidine kinase (HsvTK) vector (DesPro-TK) or a control vector (DesPro) (Fig. 2a), and treated the tumor-bearing mice with ganciclovir (GCV) to genetically deplete GSC-derived pericytes in GBM xenografts. Fifteen days after tumor implantation, mice were treated with TMZ for 3 continuous days together with or without GCV for 4 days (Fig. 2b). In vivo bioluminescence analysis revealed that pericyte depletion by GCV markedly improved the efficacy of TMZ (Fig. 2c, d). Interestingly, GCV treatment alone showed minimal influence on tumor growth (Fig. 2c, d), suggesting that pericytes may exert a supportive effect through protecting GBM cells from TMZ-induced apoptosis rather than promoting GBM proliferation. We next determined whether pericyte depletion could combine with TMZ chemotherapy to improve animal survival. Survival analysis revealed that the combined treatment of GCV and TMZ extended the survival of mice bearing control xenografts from 26.2 to 34.2 days, and significantly prolonged the survival of mice bearing DesPro-TK xenografts from 26.8 to 44.0 days (Fig. 2e). Immunofluorescence staining confirmed that GCV administration markedly impaired pericyte survival in the DesPro-TK xenografts relative to that in the DesPro xenografts (Fig. 2f, g). To investigate whether pericyte depletion increases the anti-tumor efficacy of TMZ, we determined the level of phosphorylated histone H2A variant (γ-H2AX), a DNA damage marker, in GBM xenografts. We observed an increased proportion of γ-H2AX-positive cells in TMZ-treated DesPro-TK xenografts relative to the TMZ-treated DesPro xenografts (Fig. 2h, i). In addition, we performed the above animal experiments in pericyte^low^ GBM xenografts. GSC-derived pericytes were effectively depleted in GBM-3 (pericyte^low^) DesPro-TK xenografts as observed by immunofluorescence staining of Desmin (Supplementary information, Fig. S2a, b). Survival analysis revealed that TMZ treatment extended the survival of tumor-bearing mice in both DesPro and DesPro-TK xenografts, but the combination of TMZ and GCV in pericyte^low^ GBM xenografts did not prolong the survival of mice bearing DesPro-TK xenografts (Supplementary information, Fig. S2c). Furthermore, the proportion of γ-H2AX-positive cells was similar in DesPro and DesPro-TK xenografts with TMZ treatment (Supplementary information, Fig. S2d, e), indicating that depleting pericytes in pericyte^low^ GBM xenografts had minimal influence on improving the anti-tumor efficacy of TMZ. These data demonstrate that the tumor-protective effect of pericytes on TMZ-treated GBM xenografts is largely dependent on the proportion of GSC-derived pericytes in GBM xenografts.

To determine whether the protective effects of pericytes on GBM cells from TMZ is related to pericyte coverage-related drug penetration,^10,24^ we examined TMZ half-life in murine blood and intracranial xenografts by liquid chromatography tandem mass spectrometry (LC-MS/MS). Eliminating GSC-derived pericytes in DesPro-TK xenografts had negligible impact on TMZ delivery in murine blood and intracranial xenografts (Fig. 2j), indicating that pericyte depletion did not influence TMZ penetration into the established tumors. In agreement with our results, previous studies have shown that TMZ is a brain-penetrating agent that could efficiently penetrate the BBB and BTB in human GBMs.^25^ Therefore, the drug effusion efficacy of TMZ into tumors may not play a major role in chemo-cytotoxicity enhancement of TMZ upon pericyte disruption.

### Isolation and identification of CD146^+^ pericytes from human GBMs

**Fig. 3:**
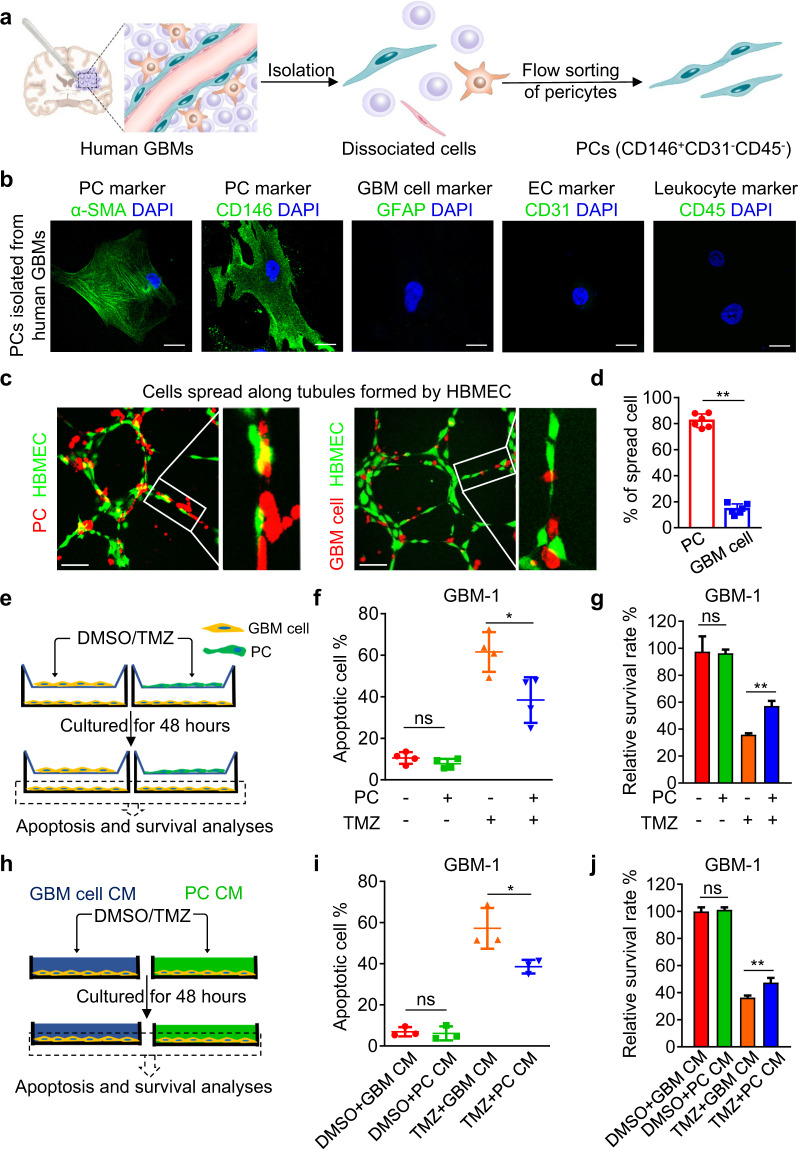
CD146^+^ pericytes isolated from human GBMs protect GBM cells from TMZ-induced apoptosis. **a** Schematic diagram of pericyte isolation from human GBMs. CD146 was used as a pericyte marker for FACS and the isolated CD146^+^ pericytes were excluded from potential contamination of endothelial cells (marked by CD31) or leukocytes (marked by CD45). **b** Immunofluorescence staining of the pericyte (PC) markers α-SMA and CD146, GBM cell marker GFAP, endothelial cell (EC) marker CD31 and leukocyte marker CD45 in CD146^+^ cells sorted from human GBMs. Scale bars, 25 μm. **c**, **d** Representative images (**c**) and morphometric analysis (**d**) of cell spreading of pericytes (red, left panel of **c**) or GBM cells (red, right panel of **c**) co-cultured with HBMECs (green). **P < 0.01. Scale bars, 50 μm. **e** Schematic diagram of transwell co-culture of pericytes and GBM cells with TMZ treatment. Human pericytes or GBM cells were added into the upper or lower chamber of transwells, respectively. TMZ or DMSO was added into the upper chamber 24 h after co-culture. Apoptosis and cell survival analyses were performed 48 h after the treatment. **f**, **g** Apoptosis (**f**) and cell survival (**g**) of GBM-1 cells with or without pericyte co-culture and TMZ treatment. ns, not significant. *P < 0.05; **P < 0.01. **h** Schematic diagram of GBM cells cultured in pericyte CM or control GBM cell CM followed by TMZ or DMSO treatment. **i**, **j** Apoptosis (**i**) or cell survival (**j**) of GBM-1 cells with or without pericyte CM stimulation and TMZ treatment. ns, not significant. *P < 0.05; **P < 0.01. Experiments in **c**–**j** were independently performed three times.

To interrogate the function of pericytes in regulating GBM cell response to TMZ, we isolated pericytes in human GBMs through fluorescence-activated cell sorting (FACS) using the well-characterized pericyte marker CD146^22^ (Fig. 3a). A CD146 antibody was generated to specifically recognize the extracellular domain of CD146, the sensitivity of which was confirmed by enzyme linked immunosorbent assay (ELISA) (Supplementary information, Table S1). The specificity of this CD146 antibody to recognize pericytes in GBMs was validated by immunofluorescence staining, showing that the CD146^+^ cells were also positive for the pericyte marker platelet derived growth factor receptor β (PDGFRβ), and were tangentially located to the outer surfaces of the endothelial tubes marked by CD31 (Supplementary information, Fig. S3a). We used this CD146 antibody to isolate pericytes from human GBMs, and excluded the possible contamination of endothelial cells (CD31^+^) and leukocytes (CD45^+^) via FACS by sorting out the CD146^+^CD31^–^CD45^–^ cell population (Fig. 3a; Supplementary information, Fig. S3b). Immunofluorescence analysis confirmed that the isolated CD146^+^CD31^–^CD45^–^ pericytes from human GBMs expressed pericyte markers α-smooth muscle actin (α-SMA), CD146 and PDGFRβ, but did not express GBM cell marker glial fibrillary acidic protein (GFAP), endothelial cell marker CD31, leukocyte marker CD45 or macrophage marker CD68 (Fig. 3b; Supplementary information, Fig. S3c). Functionally, the isolated CD146^+^CD31^–^CD45^–^ pericytes were well stretched over vascular tubules formed by human brain microvascular endothelial cells (HBMECs), and the proportion of stretched cells from pericytes (82.2% ± 2.2%) was significantly higher than that from control GBM cells (14.3% ± 1.6%) (Fig. 3c, d). Through these approaches, we validated the cellular identity of CD146^+^ pericytes isolated from human GBMs.

### CD146^+^ pericytes protect GBM cells from the TMZ-induced cytotoxicity

To assess how pericytes regulate GBM cell response to TMZ, we co-cultured primary GBM cells (GBM-1 or GBM-2) and CD146^+^ pericytes isolated from human GBMs in the lower or upper chambers of transwells and added TMZ or dimethyl sulfoxide (DMSO) to the upper chamber (Fig. 3e). As revealed by apoptotic response to TMZ, pericyte co-culture significantly reduced the percentage of apoptotic GBM-1 cells upon TMZ treatment to 38.5%, compared to 61.6% apoptotic cells in the control group (Fig. 3f). Likewise, the proportion of surviving GBM-1 cells upon TMZ treatment was markedly increased with pericyte co-culture (Fig. 3g). The tumor-protective effect of pericytes upon TMZ treatment was confirmed by a co-culture model using pericytes and GBM-2 cells (Supplementary information, Fig. S3d, e). To determine whether pericytes could protect GBM cells from TMZ-induced DNA damage, we evaluated the level of γ-H2AX and found that γ-H2AX level in GBM cells co-cultured with pericytes was dramatically reduced in comparison with control GBM cells upon TMZ treatment (Supplementary information, Fig. S3f). To determine whether pericytes exert their supportive effects through paracrine stimulation, we treated GBM cells with conditioned medium (CM) from pericytes or control GBM cells (Fig. 3h). The anti-apoptotic effects of pericytes on GBM cells upon TMZ treatment could be mimicked by pericyte CM but not by GBM cell CM (Fig. 3i, j; Supplementary information, Fig. S3g, h). Taken together, these results demonstrate that pericytes protect GBM cells from TMZ-induced DNA damage and cytotoxicity in a cell non-autonomous manner.

### Pericytes preferentially secrete CCL5 to constitute a paracrine signaling with CCR5 expressed on GBM cells

**Fig. 4:**
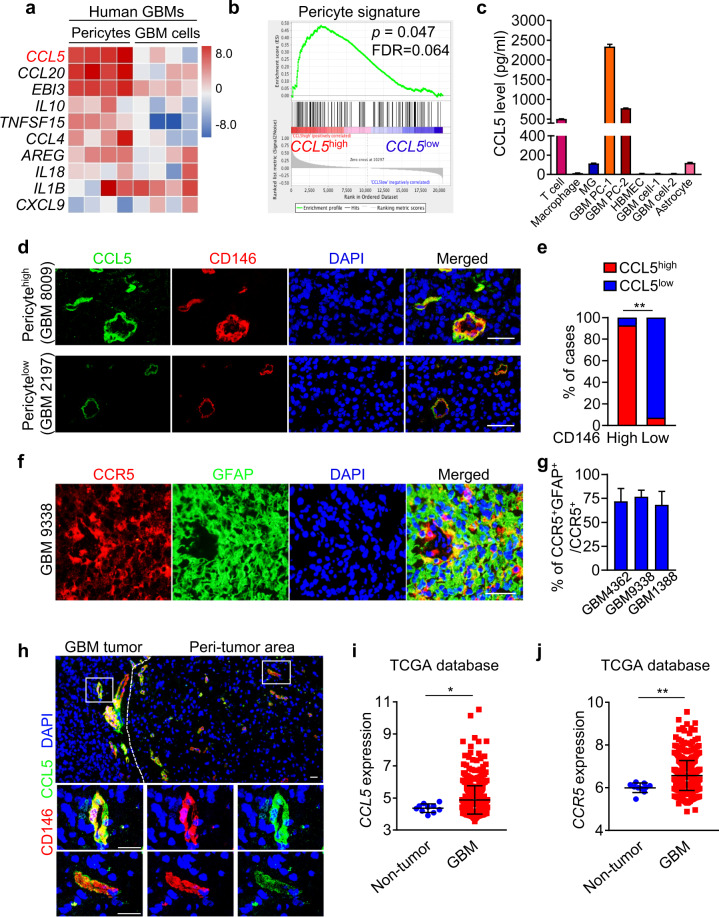
CCL5-CCR5 paracrine axis represents a molecular link between pericytes and GBM cells. **a** Top 10 upregulated cytokines in human pericytes relative to GBM cells identified by RNA-Seq and qRT-PCR analyses. **b** GSEA of pericyte score in human GBMs from the TCGA database (*n* = 152). **c** ELISA of CCL5 level in T cell, macrophage, microglia (MG), GBM pericytes (GBM PC-1, -2), HBMECs, GBM cells (GBM cell-1, -2) and astrocyte. Expression of CCL5 in CD3^+^ T cells was used as a positive control. **d**, **e** Immunofluorescence staining (**d**) and correlation (**e**) of CCL5 (green) and CD146 (red) expressions in human GBMs (*n* = 28). Scale bars, 50 μm. **P < 0.01. **f**, **g** Immunofluorescence staining of CCR5 (red) and GFAP (green) (**f**) and proportion of CCR5^+^/GFAP^+^ GBM cells in CCR5^+^ cells (**g**) in human GBMs. Scale bars, 50 μm. **h** Immunofluorescence staining of CD146 (red) and CCL5 (green) in GBM tumor and peri-tumor area of GBM. Scale bars, 25 μm. **i**, **j** Expression of *CCL5* (**i**) and *CCR5* (**j**) in human GBMs (*n* = 528) and non-tumor tissues (*n* = 10) from the TCGA database. *P < 0.05; **P < 0.01. Experiments were performed three times independently (**a**, **c**).

To identify potential factors mediating the protective effect of pericytes on GBM cells from TMZ, we screened cytokines preferentially secreted by pericytes in human GBMs. Genes upregulated in pericytes (CD146^+^CD31^–^CD45^–^) relative to the control cells (CD146^–^CD31^–^CD45^–^) in human GBMs were identified by RNA sequencing (RNA-Seq). The 517 upregulated genes in pericytes were compared with the human cytokine gene ontology set (GO_CYTOKINE_ACTIVITY, M19159), and 18 cytokine-encoding genes were identified to be preferentially expressed by pericytes (Supplementary information, Table S2). Strikingly, CCL5 was the most upregulated cytokine in pericytes relative to the control GBM cells, as confirmed by quantitative real-time PCR (qRT-PCR) analyses (Fig. 4a; Supplementary information, Table S2). Furthermore, gene set enrichment analysis (GSEA) revealed that genes associated with pericyte signature were markedly enriched in the CCL5^high^ cells relative to the CCL5^low^ cells (Fig. 4b). The correlation between *CCL5* and the pericyte markers (*Desmin* (*DES*), *platelet derived growth factor receptor beta* (*PDGFRB*) or *actin alpha 2* (*ACTA2*)) was confirmed in both the TCGA-GBM and the Chinese Glioma Genome Atlas (CGGA)-GBM datasets (Supplementary information, Fig. S4a, b). To address whether CCL5 was predominately produced by pericytes rather than other cellular components in GBMs, we measured CCL5 in the supernatants of human pericytes, immune cells (T cells, macrophages, microglias), HBMECs, GBM cells and astrocytes, and found that the pericyte population was the dominant source of CCL5 in human GBMs (Fig. 4c). Immunofluorescence staining confirmed that CCL5 was predominantly expressed by CD146^+^ pericytes that wrapped around the CD31^+^ endothelial tubing of blood vessels in human GBMs (Fig. 4d; Supplementary information, Fig. S4c). Moreover, tumor areas with high levels of CD146^+^ pericytes showed intense CCL5 staining whereas the areas with sparse CD146^+^ pericytes showed scarce CCL5 staining (Fig. 4d, e). To determine how CCL5 interacts with GBM cells, we examined the expression of CCR5, an important CCL5 receptor, in human GBMs. Immunofluorescence analysis showed that CCR5 was highly expressed in GBM cells marked by GFAP (Fig. 4f, g). To interrogate the clinical relevance of CCL5 and CCR5, we analyzed their expressions in human GBM frozen sections and GBMs in TCGA/CGGA datasets and found that both CCL5 and CCR5 were highly expressed in human GBMs relative to normal brain tissues (Fig. 4h–j; Supplementary information, Fig. S4d, e), suggesting that the CCL5-CCR5 axis may act as a potential therapeutic target for GBM treatment. Taken together, our results indicate that pericyte-derived CCL5 and its receptor CCR5 expressed on GBM cells, may constitute a paracrine axis to mediate protection of GBM cells by pericytes from TMZ-induced cytotoxicity.

### The CCL5-CCR5 signaling axis potentiates TMZ resistance of GBM cells through activating DDR signaling

**Fig. 5:**
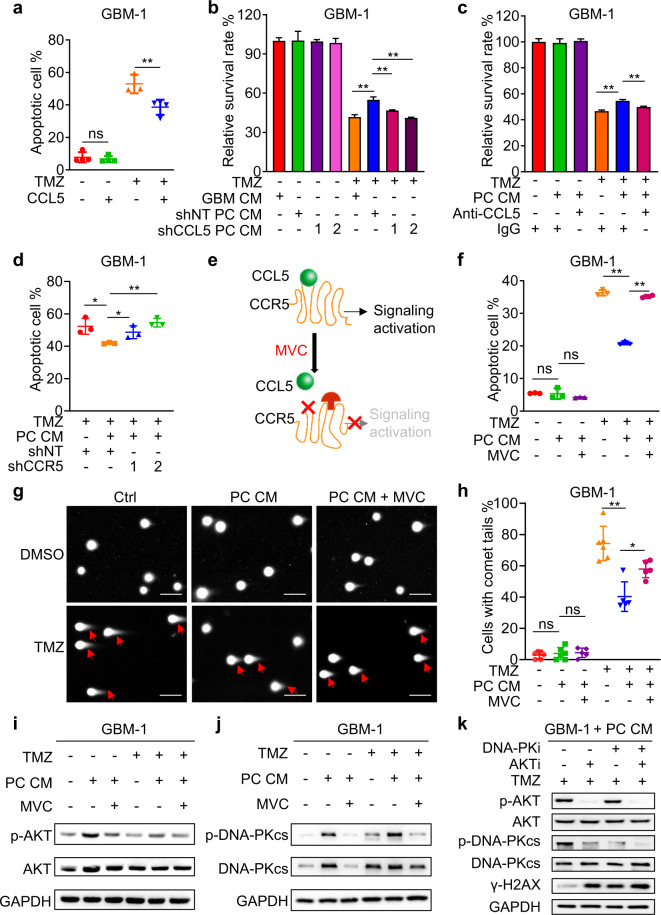
CCL5-CCR5 paracrine signaling activates DDR signaling in GBM cells to potentiate TMZ resistance. **a** Apoptosis analysis of GBM-1 cells with indicated treatments. GBM cells were stimulated with CCL5 (10 ng/mL) or vehicle (PBS) followed by the administration of TMZ (500 μmol/L) or DMSO. ns, not significant. **P < 0.01. **b** Cell survival analysis of GBM-1 cells with indicated treatments. CM from pericytes expressing shNT or shCCL5 (sh-1 or sh-2) was collected and added to GBM cells. GBM cells were treated with TMZ (500 μmol/L) after CM addition. **P < 0.01. **c** Cell survival analysis of GBM-1 cells with indicated treatments. Pericyte CM was pretreated with neutralizing anti-CCL5 antibody or IgG before being added to GBM cells. GBM cells were treated with TMZ (500 μmol/L) after pericyte CM addition. **P < 0.01. **d** Apoptosis analysis of GBM-1 cells with indicated treatments. GBM cells expressing shNT or shCCR5 (sh-1 or sh-2) were pretreated with pericyte CM or control GBM cell CM followed by TMZ (500 μmol/L) treatment. *P < 0.05; **P < 0.01. **e** Schematic diagram of MVC-mediated inhibition of CCL5-CCR5 signaling. The binding of MVC to CCR5 leads to a conformational change of its extracellular domain to prevent ligand-stimulated CCR5 activation. **f** Apoptosis analysis of GBM-1 cells with indicated treatments. GBM-1 cells were pretreated with MVC (500 nmol/L) or DMSO for 1 h, followed by pericyte CM stimulation. Apoptosis and cell survival analyses were performed 48 h after TMZ treatment. ns, not significant. **P < 0.01. **g** DNA damage after the indicated treatments was assessed by comet assay. Scale bars, 50 μm. **h** Quantification of percentage of cells with comet tails with indicated treatments. ns, not significant. *P < 0.05; **P < 0.01. **i**, **j** Immunoblot analysis of phosphorylated AKT (Ser473) and AKT (**i**) or phosphorylated DNA-PKcs (Ser2056) and DNA-PKcs (**j**) in GBM-1 cells with indicated treatments. **k** Immunoblot analysis of phosphorylated AKT (Ser473), AKT, phosphorylated DNA-PKcs (Ser2056), DNA-PKcs, γ-H2AX in GBM-1 cells with indicated treatments. DNA-PKi (DNA-PKcs inhibitor) represents KU-57788; AKTi (AKT inhibitor) represents MK-2206. Experiments in **a**–**k** were independently performed three times.

To address whether CCL5-CCR5 signaling mediates the protective effect of pericytes against TMZ, GBM-1 cells were pretreated with recombinant human CCL5 (10 ng/mL) or phosphate buffer saline (PBS) followed by exposure to TMZ or DMSO. We found that CCL5 stimulation reduced apoptosis and maintained survival of GBM-1 cells treated with TMZ (Fig. 5a; Supplementary information, Fig. S5a), indicating that CCL5 mimicked the protective effect of pericytes. Disrupting CCL5 expression in pericytes by shRNAs (shCCL5-1 or shCCL5-2) (Supplementary information, Fig. S5b) significantly compromised the protective effect of pericyte CM as compared to control CM from pericytes expressing non-targeting shRNA (shNT) (Fig. 5b; Supplementary information, Fig. S5c). Blockade of CCL5 by administration of neutralizing antibody also partially impaired the protective effect of pericytes on GBM cells from TMZ (Fig. 5c; Supplementary information, Fig. S5d). We next determined whether pericytes function through stimulating CCR5 expressed on GBM cells. To this end, we established GBM cells expressing shCCR5 or shNT (Supplementary information, Fig. S5e), which were treated with pericyte CM followed by TMZ treatment. Analyses of apoptosis and cell survival showed that CCR5 disruption markedly inhibited the protective effect of pericytes on GBM viability upon TMZ treatment (Fig. 5d; Supplementary information, Fig. S5f, g). To determine whether blocking the CCL5-CCR5 axis would inhibit the supportive effects of pericytes, we employed the CCR5 antagonist MVC, which binds to CCR5 leading to a conformational change to prevent ligand-stimulated CCR5 signaling activation (Fig. 5e).^26^ Treatment with MVC inhibited the protective effects of pericyte CM on TMZ-induced cell apoptosis (Fig. 5f) and TMZ-impaired cell survival (Supplementary information, Fig. S5h). Comet tail analysis demonstrated that TMZ-induced DNA damage of GBM cells was significantly rescued by pericyte CM stimulation, whereas MVC treatment impaired the effect of pericyte CM (Fig. 5g, h). Consistently, immunoblot analysis of γ-H2AX confirmed that disrupting CCL5-CCR5 axis by MVC effectively blocked the protection from TMZ-induced DNA damage by pericytes (Supplementary information, Fig. S5i). These results indicate that the CCL5-CCR5 axis is a critical molecular link mediating the protective effect of pericytes on GBM cells against TMZ treatment.

We next assessed the downstream cascade mediating CCL5-CCR5 signal transduction in GBM cells. The AKT pathway, the overactivation of which is frequently observed in TMZ-resistant GBMs,^27^ is associated with CCR5 signaling.^28^ We found that CCL5 stimulation activated AKT as monitored by phosphorylation of AKT on Serine 473, whereas addition of the AKT inhibitor MK-2206 significantly abrogated CCL5-mediated AKT activation (Supplementary information, Fig. S5j). Likewise, pericyte CM-triggered AKT activation was largely abrogated by CCR5 antagonist MVC (Fig. 5i). To address how the AKT pathway mediates CCL5-CCR5 signaling to inhibit TMZ-induced DNA damage, we examined the activation of DNA-PKcs, an important DDR effector downstream of AKT.^29^ Immunoblot analysis revealed that disruption of CCL5-CCR5 signaling via MVC markedly abrogated pericyte CM-induced activation of DDR upon TMZ treatment (Fig. 5j). Moreover, treatment of the AKT inhibitor MK-2206, or the DNA-PKcs inhibitor KU-57788, could abrogate pericyte CM-activated DDR upon TMZ treatment, as shown by reduced phosphorylation of DNA-PKcs and increased γ-H2AX in GBM cells treated with each inhibitor (Fig. 5k; Supplementary information, Fig. S5k). Meanwhile, combined treatment of MK-2206 and KU-57788 on GBM cells cultured with pericyte CM did not show any increased effect on DDR (Fig. 5k; Supplementary information, Fig. S5k), suggesting that AKT and DNA-PKcs may function together in a signaling cascade to mediate DDR upon activation by pericyte CM. Collectively, these results indicate that the CCL5-CCR5 signaling could activate the AKT-DNA-PKcs pathway to potentiate DDR, thus impairing the cytotoxic effect of TMZ on GBM cells.

### The CCR5 antagonist MVC effectively combines with TMZ to impair GBM growth

**Fig. 6:**
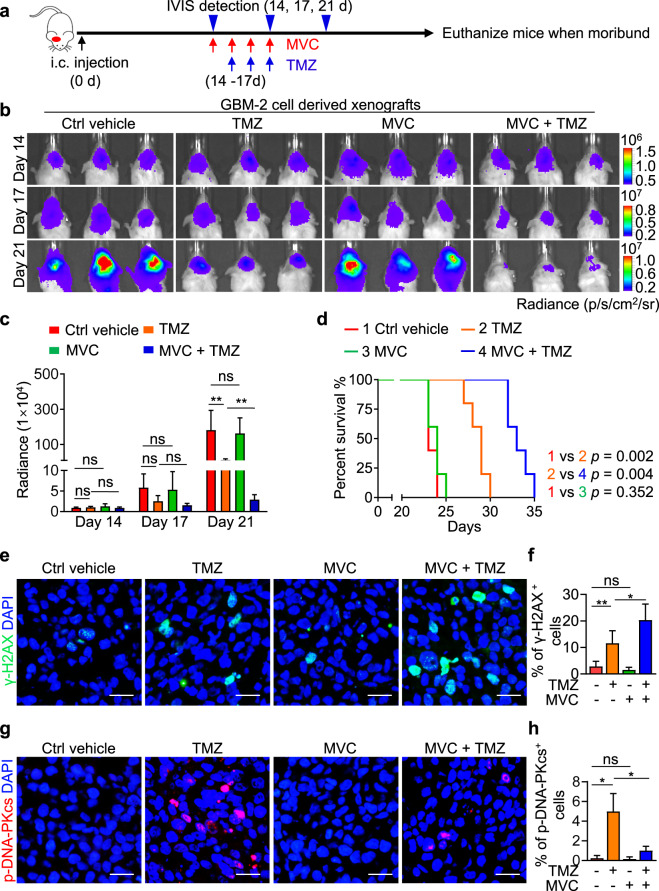
MVC, as a CCR5 antagonist, combines with TMZ to effectively impair GBM growth. **a** Schematic diagram of the combined treatment of MVC and TMZ in tumor-bearing mice. MVC (100 mg/kg, i.p.) was given for 4 consecutive days since Day 14 and TMZ (5 mg/kg, i.p.) was given for 3 consecutive days since Day 15 after tumor implantation. The growth of GBM-2 xenografts was monitored by bioluminescence imaging on Day 14, Day 17 and Day 21 after tumor implantation. **b**, **c** In vivo bioluminescence images (**b**) and quantification (**c**) of tumor growth of GBM-2 xenografts treated with TMZ, MVC or vehicle on Day 14, Day 17 and Day 21 after tumor implantation. ns, not significant. *P < 0.05; **P < 0.01. *n* = 5 for each group. **d** Kaplan–Meier survival analysis of mice bearing GBM-2 xenografts treated with TMZ, MVC or vehicle. *n* = 5 for each group. **e**, **f** Immunofluorescence staining (**e**) and quantification (**f**) of γ-H2AX-positive cells (green) in GBM-2 xenografts treated with TMZ, MVC or vehicle. ns, not significant. *P < 0.05; **P < 0.01. Scale bars, 25 μm. **g**, **h** Immunofluorescence staining (**g**) and quantification (**h**) of phosphorylated DNA-PKcs (Ser2056, red) in GBM-2 xenografts treated with TMZ, MVC or vehicle. ns, not significant. *P < 0.05. Scale bars, 25 μm.

To explore whether MVC administration could enhance TMZ therapeutic efficacy in vivo, we established primary GBM xenografts using GBM-2 cells expressing luciferase. Fourteen days after tumor cell implantation, mice were treated with TMZ (5 mg/kg, i.p., for 3 consecutive days) together with or without MVC (100 mg/kg, i.p., for 4 consecutive days) or the control vehicle, and tumor growth was monitored by bioluminescence signal using an In Vivo Imaging System (IVIS) (Fig. 6a). Bioluminescence analysis revealed that while MVC treatment alone exerted minimal influence on the growth of GBM-2 xenografts (Fig. 6b, c), combined treatment of MVC and TMZ significantly enhanced the tumor-suppressive effect of TMZ, as shown by the 75.2% reduction of the bioluminescence signal in xenografts treated with MVC and TMZ relative to those treated with TMZ alone on Day 21 (Fig. 6b, c). Consistent with this effect on tumor growth, the Kaplan–Meier survival analysis showed that MVC treatment alone exhibited negligible impact on animal survival (Fig. 6d). However, mice treated with MVC and TMZ conferred 1.88-fold survival extension relative to those treated with TMZ alone (Fig. 6d), confirming that the addition of MVC enhanced the therapeutic efficacy of TMZ to suppress GBM growth. We next determined the molecular basis underlying the effect of MVC on GBM xenografts. Xenografts treated with MVC and TMZ showed substantially increased DNA damage (marked by γ-H2AX) relative to those treated with TMZ alone (Fig. 6e, f). Moreover, the addition of MVC suppressed DDR in GBM xenografts treated with TMZ, as revealed by the reduced activation of DNA-PKcs in xenografts treated with MVC and TMZ relative to those treated with TMZ alone (Fig. 6g, h). MVC treatment had no effect on pericyte coverage in xenografts (Supplementary information, Fig. S6a, b), suggesting that MVC inhibits the CCL5-CCR5 pathway but does not influence pericyte survival. Collectively, these results demonstrate that MVC is a CCR5 antagonist that effectively enhances the therapeutic efficacy of TMZ against GBM.

### Increased CCL5 informs poor therapeutic efficacy of TMZ and predicts worse outcome of GBM patients

**Fig. 7:**
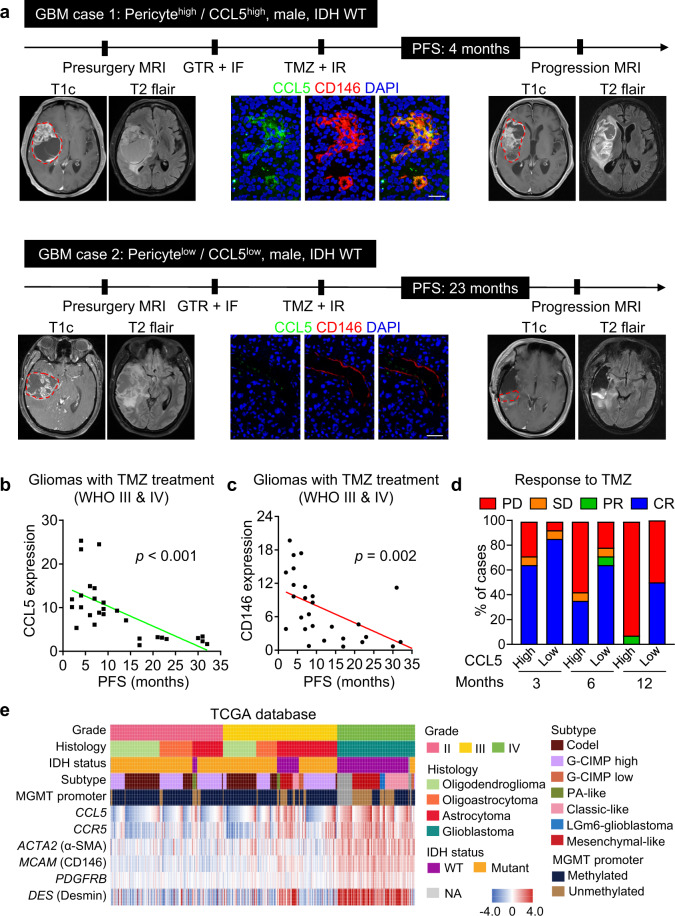
Increased CCL5 informs poor therapeutic efficacy of TMZ and worse outcome of GBM patients. **a** Therapeutic response of TMZ in two representative GBM patients with high or low CCL5 expression. Representative MRI, immunofluorescence staining of CD146 (red) and CCL5 (green) and PFS were shown. Tumor border was marked by red dotted lines in enhanced T1 image (T1c) of MRI. Patients with low CCL5/pericyte proportion benefit more from TMZ treatment relative to those with high CCL5/pericyte proportion. Scale bars, 50 μm. **b**, **c** Correlation analysis of CCL5 level (**b**) or CD146 level (**c**) and PFS of glioma patients (*n* = 28). High level of CCL5 or CD146 indicates rapid glioma recurrence. **d** Therapeutic response to TMZ in glioma patients with high or low CCL5 expression (*n* = 28). Therapeutic responses were evaluated by the Response Assessment in Neuro-Oncology (RANO) standard. CR, complete response; PR, partial response; PD, progressive disease; SD, stable disease. **e** Expression patterns of *CCL5*, *CCR5* and pericyte markers *ACTA2*, *MCAM*, *PDGFRB* and *DES* in human gliomas with different molecular signatures using the TCGA pan-glioma (GBM-LGG) dataset (*n* = 669). Codel, co-deletion of chromosomes 1p and 19q; G-CIMP, glioma-CpG island methylator phenotype (CpG, C-phosphate-G); PA-like, pilocytic astrocytoma-like; LGm6-glioblastoma, a subgroup of glioma enriched for histologic low-grade gliomas but also contains a subset of tumors with GBM-defining histologic criteria; MGMT, O^6^-methylguanine-DNA methyltransferase; IDH, isocitrate dehydrogenase; WT, wild type; NA, not available.

Given the pivotal role of pericyte-derived CCL5 in mediating TMZ resistance, we evaluated whether CCL5 could be used as a biomarker to predict TMZ response in human GBMs. A cohort of 28 cases of primary glioma patients who underwent complete tumor resection and standard TMZ treatment were recruited. Tumor recurrent status was monitored via magnetic resonance imaging (MRI) and symptom evaluation. The expression of CCL5 in CD146^+^ pericytes and tumor recurrence status of two representative isocitrate dehydrogenase (IDH)-wild-type GBM patients (Case 1: CCL5^high^/CD146^high^ and Case 2: CCL5^low^/CD146^low^) were assessed. We found that high levels of CCL5 produced by pericytes correlated with short progression-free survival (PFS) and rapid tumor recurrence after TMZ treatment, whereas tumors with low levels of CCL5 correlated with longer PFS and slower tumor recurrence after TMZ treatment (Fig. 7a–d), suggesting that CCL5 could effectively predict TMZ efficacy in gliomas. We further analyzed a TCGA-GBM dataset of 312 cases of GBMs with follow-up information. The univariate and multivariate Cox-regression analyses revealed that CCL5 was an independent prognostic marker informing poor outcomes of GBM patients (Supplementary information, Table S3). To interrogate the translational significance of CCL5-CCR5 signaling in gliomas, we investigated a low-grade glioma (LGG)-GBM cohort from TCGA database with tumor grades, histology, subtypes and O^6^-methylguanine-DNA-methyltransferase (MGMT) promoter methylation information. Preferential expression of *CCL5* or *CCR5* was identified in GBMs, IDH-wild-type gliomas and mesenchymal-like subtype gliomas (Fig. 7e). No association between *CCL5* or *CCR5* expression and MGMT promoter methylation was observed (Fig. 7e). The expression patterns of pericyte markers (*ACTA2*, *melanoma cell adhesion molecule* (*MCAM*), *PDGFRB* and *DES*) and their clinical significance were similar to that of *CCL5* (Fig. 7e). These results demonstrate that increased CCL5 signifies poor TMZ therapeutic efficacy and worse clinical outcome of GBMs.

### GBMs with high CCL5 expression benefit from combined treatment of TMZ and MVC

**Fig. 8:**
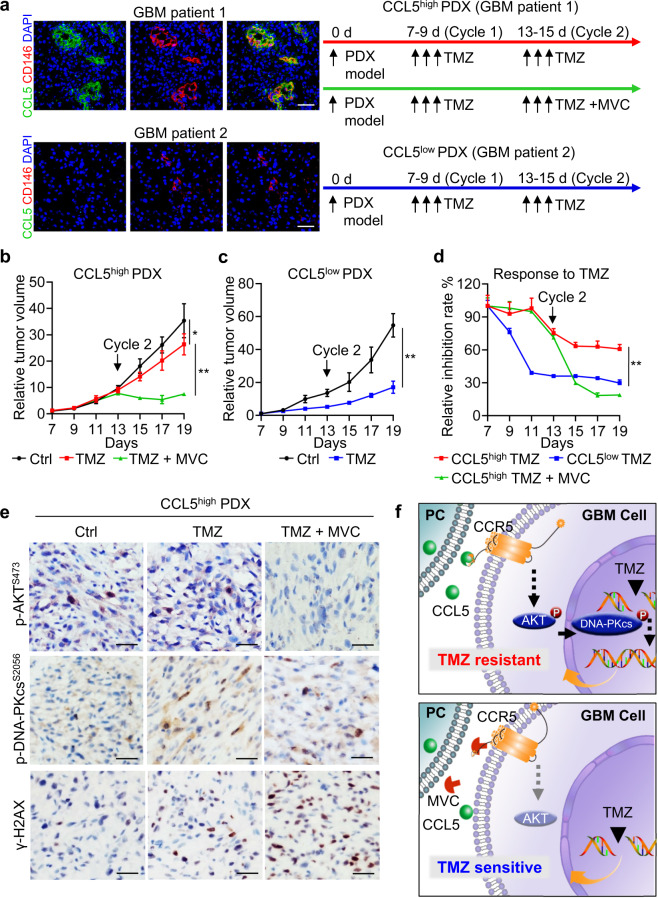
GBM PDXs with high CCL5 expression benefit from combined treatment of TMZ and MVC. **a** Development of adjuvant MVC treatment together with TMZ chemo-treatment according to CCL5 expression in GBM PDXs. PDXs were cultured and passaged in NCG mice in vivo. Representative immunofluorescence staining of CCL5 (green) and CD146 (red) in two human GBMs (CCL5^high^ or CCL5^low^) with PDX model constructed. CCL5^high^ PDXs were treated with two cycles of TMZ (5 mg/kg, i.p., 3 consecutive days per cycle starting from Day 7 and Day 13) and one cycle of MVC (100 mg/kg, i.p., 3 consecutive days starting from Day 13), while CCL5^low^ PDXs were treated with two cycles of TMZ (5 mg/kg, i.p., 3 consecutive days per cycle starting from Day 7 and Day 13) alone. Scale bars, 50 μm. **b**, **c** Relative tumor volume of CCL5^high^ PDXs (**b**) and CCL5^low^ PDXs (**c**) with indicated treatments. *n* = 5 for each group. *P < 0.05; **P < 0.01. **d** Relative inhibition rate of CCL5^high^ PDXs treated with TMZ (red), TMZ + MVC (green) and CCL5^low^ PDXs treated with TMZ (blue). **P < 0.01. **e** Representative images of immunohistochemistry staining of phosphorylated AKT (Ser473), phosphorylated DNA-PKcs (Ser2056) and γ-H2AX (Ser139) in CCL5^high^ PDXs treated with TMZ together with or without MVC as an adjuvant agent. Scale bars, 50 μm. **f** Schematic diagram of the mechanism underlying pericyte-mediated DDR and TMZ resistance.

To provide a translational rationale of exploiting MVC as an adjuvant agent to improve GBM chemotherapy, we developed a personalized therapeutic approach using TMZ and MVC according to CCL5 expression in primary GBM tissues. Patient-derived tumor xenografts (PDXs) were established from two human GBMs presented with high/low CCL5 expression (Fig. 8a, left panel). Typical GBM morphological features, including the hypervascular proliferation and pseudopalisading necrosis, were identified in primary GBMs and their corresponding PDXs (Supplementary information, Fig. S7a). Mice bearing CCL5^high^ PDXs were treated with one cycle of TMZ (5 mg/kg, i.p., 3 consecutive days) and one cycle of TMZ together with or without MVC (100 mg/kg, i.p., 3 consecutive days), while those bearing CCL5^low^ PDXs were treated with two cycles of TMZ (5 mg/kg, i.p., 3 consecutive days) alone (Fig. 8a, right panel). The tumor size analysis showed that TMZ treatment alone modestly inhibited the growth of CCL5^high^ PDXs (Fig. 8b), but markedly restrained the growth of CCL5^low^ PDXs (Fig. 8c), confirming the differential sensitivity to TMZ between CCL5^high^ and CCL5^low^ PDXs (Fig. 8d). Intriguingly, the administration of adjuvant MVC in the second cycle of TMZ treatment effectively impaired the growth of CCL5^high^ PDXs (Fig. 8b), as shown by a 78.8% tumor reduction in the CCL5^high^ TMZ+MVC group relative to the 25.2% reduction in the CCL5^high^ TMZ group (Fig. 8d). Moreover, the tumor inhibition rate of CCL5^high^ PDXs with TMZ+MVC was similar to that of CCL5^low^ PDXs with TMZ alone (Fig. 8d), suggesting that adjuvant MVC treatment reversed CCL5^high^ PDXs from a TMZ-resistant phenotype to a TMZ-sensitive phenotype. At the molecular level, the activation of AKT and DNA-PKcs related to TMZ resistance was largely abrogated, whereas the DNA damage marker γ-H2AX was significantly increased, when adjuvant MVC was included in the treatment of CCL5^high^ PDXs (Fig. 8e; Supplementary information, Fig. S7b). Moreover, immunohistochemistry staining of cleaved caspase-3 showed that adjuvant MVC treatment manifested no severe damage effect on spleen or lung tissue presented with CCR5 expression, nor on liver or kidney tissue metabolism (Supplementary information, Fig. S7c and Table S4). Consistent with results in Supplementary information, Fig. S6, neither MVC nor TMZ had any effect on overall pericyte number (Supplementary information, Fig. S7d, e). These preclinical data provide a rationale for using MVC as a TMZ-sensitizing agent to improve therapeutic efficacy in GBMs with high CCL5 expression (Supplementary information, Fig. S8).

In summary, our data demonstrate that pericyte-secreted CCL5 stimulates CCR5 on GBM cells, which promotes activation of the AKT-DNA-PKcs pathway to potentiate DNA repair, thus abrogating TMZ-induced GBM cell apoptosis. MVC administration blocks CCL5-CCR5 signaling and disrupts DDR, thus reversing GBM cells from a TMZ-resistant to a TMZ-sensitive phenotype (Fig. 8f).

## Discussion

Pericytes represent a specialized mesenchymal cell type within the tumor perivascular niche and potentiates tumor vascular functions, tumor metastasis and immune cell infiltration.^9,30–32^ Pericytes in GBMs exhibit excessive outgrowth^33^ and are pivotal for maintaining the integrity of the BTB.^10^ In this study, we reveal that pericytes directly interact with GBM cells residing in the perivascular niche to enhance DNA repair and induce TMZ chemoresistance. Pericytes secrete CCL5 to stimulate CCR5 that is highly expressed by GBM cells, constituting a critical paracrine signaling within the GBM perivascular niche. Silencing the CCL5-CCR5 signaling largely abrogates the tumor-protective effects of pericytes and enhances the chemotherapeutic efficacy of TMZ. Our study provides new insights into the functions of pericytes in supporting GBM progression and highlights the CCL5-CCR5 paracrine axis as a critical molecular link mediating pericyte–GBM cell interaction with therapeutic potential as a chemosensitizing target.

Our previous work demonstrated that pericyte depletion disrupted the BTB to enhance drug delivery and anti-tumor efficacy of etoposide, a poor BTB-penetrating chemoagent (the ratio of cerebrospinal spinal fluid to blood concentration for etoposide is 0.1%–0.2%).^10,34^ However, TMZ, the first-line therapy for GBM, has a satisfactory brain-penetrating efficacy,^25^ and our results reveal that pericyte-mediated DDR enhancement is a major mechanism of the effect of pericytes on TMZ resistance, which is independent of the role of pericytes in sustaining vascular integrity. In agreement with our work, recent reports indicate that β3 integrin-negative pericytes may communicate with lung cancer cells and melanoma cells to support tumor proliferation through the CCL2-MAP kinase kinase 1 (MEK1)-Rho associated coiled-coil containing protein kinase 2 (ROCK2) pathway,^21^ underscoring the multiple functions of pericytes in promoting tumor progression.

Besides the interaction with tumor cells, pericytes may protect other perivascular components including endothelial cells from chemo-cytotoxicity and mediate the therapeutic response of antiangiogenic agents and other molecular targeted therapies.^35–38^ Our results underscore the functional significance of pericytes in sustaining a favorable niche for tumor survival under therapeutic conditions. Considering that the origin of pericytes and its therapy-driven evolution in GBMs during chemo-treatment remain unclear, further studies remain necessary regarding aspects of pericyte signatures both in pre- and post-treatment lesions to investigate their roles in mediating the acquired resistance to tumor therapies.

Enhanced DDR represents a major impediment to effective chemo-treatment. Recent studies imply that DNA repair should not be simply regarded as an intrinsic property of tumor cells, but a biological process that could be activated by environmental cues.^39^ Together with previous studies, our results unveil that hyperactivated CCL5-CCR5 signaling governs DNA repair activation in tumors.^28^ In the context of GBM, pericyte-secreted CCL5 binds to CCR5 expressed on GBM cells triggering the activations of AKT and downstream DNA-PKcs to promote DDR, thus overcoming the TMZ-induced cytotoxicity. Of note, CCL5-CCR5 signaling-mediated DNA repair is irrelevant to the expression of DNA repair enzyme MGMT in GBM cells. In line with our findings, the overactivation of CCL5-CCR5 signaling and their oncogenic effects on tumor malignant behaviors have been reported in other cancers.^28,40,41^ These results underlie the importance of targeting the CCL5-CCR5 axis for tumor treatment.

In the era of precision medicine, the employment of appropriate targeted therapies according to individualized tumor signatures have emerged as an attractive therapeutic approach.^42,43^ Based on previous studies that MVC improves the efficacy of DNA damage agents,^28,44^ we proposed a therapeutic strategy of combining CCL5-CCR5 targeting agents such as MVC with TMZ to improve chemotherapeutic efficacy. Our preclinical data indicate that adjuvant MVC treatment acts as a chemosensitizing agent and reverses CCL5^high^ GBM from a TMZ-resistant phenotype to a TMZ-sensitive phenotype. Emerging clinical trials show promising anti-tumor activity of CCR5 antagonists in multiple tumors (ClinicalTrials.gov Identifiers NCT01736813, NCT03274804, NCT01276236, NCT03838367).^19,45^ It has been reported that MVC has a favorable safety profile, is well tolerated, and is a BBB-penetrable small-molecule antagonist of CCR5.^19,20^ Furthermore, previous studies reported that MVC inhibits tumor invasion and metastasis, suggesting that MVC possesses additional anti-tumor effects besides enhancing DNA damage agent efficacy.^46^ Therefore, repurposing MVC may represent a promising targeting approach for the treatment of malignant gliomas and other tumors with CCR5 overactivation.

## Materials and methods

### Primary glioma specimens

Human GBM surgical specimens were obtained from the Biobank of Southwest Hospital, Third Military Medical University (TMMU). All human specimens used in this study were approved by the ethics committees of TMMU, with informed consents from patients or their guardians. Histopathological diagnoses of glioma specimens were performed by two neuropathologists according to the 2016 World Health Organization (WHO) classification. Frozen and the formalin-fixed, paraffin-embedded glioma sections were stored at –20 °C or at room temperature, respectively. All procedures were performed in accordance with the principles of the Helsinki Declaration.

### Cell isolation and culture

Human GBM specimens were dissociated using Papain Dissociation system (LK003150, Worthington Biochemical Corporation) according to the manufacturer’s instructions. The isolated cell mixture was resuspended in HBSS (Gibco) plus Matrigel (Corning, 354277), and implanted into the 5–6-week-old female NOD/ShiltJGpt-Prkdcem26Cd52Il2rgem26Cd22/Gpt (NCG) immune-deficient mice to construct PDXs. GBM cells dissociated from PDXs or from primary GBMs were enriched for CD133^+^/CD15^+^ cells by FACS as previously described.^47^ The clinicopathological information of GBM cells used in the study is listed in Supplementary information, Table S5. GBM cells were cultured in Neurobasal-A medium (Gibco, 12349015) supplemented with B-27 (Gibco, 17504-044), 10 ng/mL EGF (Peprotech, #AF-100-15), 10 ng/mL bFGF (Peprotech, #AF-100-18B), 1× MEM Non-Essential Amino Acids (Gibco, 11140-050), 1× GlutaMAX (Gibco, 35035-061), 1× Sodium pyruvate (Gibco, 11360-070) and 1× Penicillin-Streptomycin Solution (HyClone, SV30010). Pericytes (PC-1–PC-4) dissociated from human GBMs were enriched for the CD146^+^CD45^–^CD31^–^ cells by FACS. Briefly, cells were labeled with CD146 antibody (self-made) and APC-labeled mouse secondary antibody (Invitrogen, A-21052, 1:500), FITC-conjugated anti-CD31 antibody (BD Pharmingen, 555445), PE/Cy7-conjugated anti-CD45 antibody (Biolegend, 368532) or the isotype IgG (Cell Signaling Technology, #5415) at 4 °C for 30 min. Pericytes were cultured in pericyte medium (PM, Sciencell, #1201). HBMECs purchased from Sciencell Research Laboratory (Cat# 8000) were cultured in endothelial cell medium (ECM, Sciencell, #1001). All cells utilized in in vitro experiments were cultured at 37 °C, 5% CO_2_, and were authenticated by karyotype analysis or short tandem repeat analysis and were verified to be free of mycoplasma contamination by PCR analysis.

### Cell co-culture, cell apoptosis and survival analyses

Pericytes (5 × 10^4^) and GBM cells (5 × 10^4^) were, respectively, cultured in the upper or lower chamber in 6-well transwell apparatus, followed by the treatment of TMZ (500 μmol/L, Selleckchem, S1237). To determine the protective effect of pericytes or CCL5 on GBM cells, GBM cells were pretreated with or without pericyte CM, recombinant human CCL5 (10 ng/mL, R&D), or MVC (500 nmol/L, Selleckchem, S2003), followed by TMZ (500 μmol/L) treatment 4 h later. Apoptotic analysis was performed using Annexin-V Apoptosis Detection Kit (Invitrogen, 88-8007-74) according to the manufacturer’s instructions. Cell survival analysis was performed using CCK-8 (Dojindo, CK04) as previously described.^48^

### Comet assay

The neutral comet assay was performed as previously described.^49,50^ GBM cells were cultured in control CM or pericyte CM for 4 h and then treated with TMZ (500 μmol/L). Low-melting point agarose and resuspended GBM cells were mixed and spread on agarose pretreated microscope slides. Cells on the slides were lysed by RIPA (Beyotime, P0013B) at 37 °C for 4 h and subjected to horizontal electrophoresis for 30 min in TAE buffer (pH 8.2), followed by staining with propidium iodide (PI). The presence of comet tails was assessed and quantified under fluorescent microscope (Olymous, BX53).

### ELISA assay

The indicated cells were cultured in serum-free medium and supernatants were collected after 48 h of culture. CCL5 production in supernatants was normalized by cell number and analyzed by ELISA (R&D) according to the manufacturer’s instructions.

### Immunofluorescence staining

Immunofluorescence analysis was performed as previously described.^47^ Primary antibodies included PDGFRβ (Cell Signaling Technology, #3169, 1:200), CD146 (Abcam, ab75769, 1:200), α-SMA (Cell Signaling Technology, #19245, 1:100), CD31 (Abcam, ab28364, 1:100; Cell Signaling Technology, 3528S, 1:100), CD45 (Millipore, 05-1410, 1:100), CD68 (Biolegend, 333802, 1:100), GFAP (Cell Signaling Technology, #12389, 1:200), CCL5 (Abcam, ab52562, 1:100) and CCR5 (Invitrogen, 14-1957-82, 1:100). TUNEL staining was performed according to the manufacturer’s instructions (Beyotime, C1090). The percentage of positive cells was defined as the ratio of positive cells relative to the total cells in five randomly selected fields. The percentage of positive areas was calculated using ImageJ software (National Institutes of Health, USA). Pericyte proportion relative to the microvascular density in GBMs was defined as the ratio of the number of CD146-positive pericytes and the number of CD31-positive vessels in five randomly selected high-power fields in each GBM sample. The images were acquired with a fluorescent (Olymous, BX53) or confocal (Leica, TCS SP8) microscope.

### Immunohistochemistry staining

Immunohistochemistry staining was performed using Dako REAL EnVision System according to the manufacturer’s instructions. Primary antibodies included α-SMA (Zsbio, ZM-0003, 1:400), p-AKT-Ser473 (Novus, NB100-79891, 1:150), p-DNA-PKcs-Ser2056 (Abcam, ab18192, 1:500) and γ-H2AX (Abcam, ab26350, 1:10,000). The percentage of positive cells or the percentage of positive areas was calculated in five randomly selected fields using ImageJ software.

### Lentiviral vector construction

Human CCR5-specific shRNA vectors, CCL5-specific shRNA vectors and shNT vectors were purchased from Ribobio (China) with shRNA sequences listed in Supplementary information, Table S6. The procedure for lentivirus packaging and transduction was performed as previously described.^47^ Cells stably expressing shNT, shCCR5 or shCCL5 were selected by FACS of EGFP-positive cells.

### qRT-PCR

qRT-PCR was performed as previously described.^47^ qRT-PCR was performed on a CFX96 Real-Time PCR Detection System (Bio-Rad). The primer sequences used are listed in Supplementary information, Table S7. The expression of *ACTB* (encoding β-actin) was used for normalization.

### Immunoblot analyses

Immunoblot assay was performed as previously described.^51^ GBM cells were treated with recombinant human CCL5 (10 ng/mL, R&D) together with or without the AKT inhibitor MK-2206 (Selleckchem, 500 nmol/L), with or without the DNA-PKcs inhibitor KU-57788 (Selleckchem, 1 μmol/L). To block the function of CCR5, MVC was added 1 h before pericyte CM stimulation. Antibodies used for immunoblot assays included anti-AKT (Cell Signaling Technology, #4685, 1:1000), anti-p-AKT-Ser473 (Cell Signaling Technology, #4060, 1:1000), anti-γ-H2AX (Cell Signaling Technology, #9718, 1:1000), anti-DNA-PKcs (Cell Signaling Technology, #38168, 1:1000), anti-p-DNA-PKcs-Ser2056 (Cell Signaling Technology, #68716, 1:1000), anti-GAPDH (Proteintech, #60004, 1:10,000).

### Intracranial GBM xenografts and treatment

All animal experiments were approved by the Institutional Animal Care and Use Committee of the Southwest Hospital in accordance with the Guide for the Care and Use of Laboratory Animals. In brief, two thousand primary GBM cells expressing luciferase reporter were transplanted into the right frontal lobe of NCG mice purchased from GemPharmatech Company. Tumor growth was monitored by bioluminescence imaging using IVIS Spectrum (Perkin-Elmer). Mice were sacrificed at the indicated time points or upon manifestation of neurological symptoms. To determine the effect of pericyte depletion on TMZ treatment, mice were treated with vehicle (5% DMSO + 30% PEG300 + 65% ddH_2_O, i.p.), TMZ (5 mg/kg, i.p., Selleckchem, S1237), GCV (50 mg/kg, i.p., Selleckchem, S1878), or the combination of TMZ and GCV. To examine the effect of MVC on TMZ treatment, mice were treated with vehicle (5% DMSO + 30% PEG300 + 65% ddH_2_O, i.p.), TMZ (5 mg/kg, i.p.), MVC (100 mg/kg, i.p.), or the combination of TMZ and MVC. Digested GBM samples were inoculated subcutaneously into the flanks of NCG mice to form PDXs. Clinicopathological information of PDXs is listed in Supplementary information, Table S8. Treatments of PDXs to replicate personalized therapy were carried out as follows, mice bearing CCL5^high^ PDXs were given one cycle of vehicle or TMZ (5 mg/kg, i.p.) since Day 7, and one cycle of vehicle, TMZ (5 mg/kg, i.p.), or the combination of TMZ and MVC (100 mg/kg, i.p.) since Day 13 after tumor implantation. Mice bearing CCL5^low^ PDXs were given two cycles of vehicle or TMZ (5 mg/kg, i.p.) since Day 7 and Day 13 after tumor implantation. Xenograft sizes were measured using automated caliper every 2 days. Mice were euthanized when tumors reached 15 mm in diameter.

### Bioinformatic analyses of human GBMs from the TCGA database

The gene expression in human GBMs with follow-up information was analyzed using gene profiling data (AffyU133a, AgilentG4502A, IlluminaHiSeq, GBMLGG dataset) from the TCGA database (https://tcga-data.nci.nih.gov/tcga) and the CGGA database (http://www.cgga.org.cn/). Pericyte score and tumor progression score were calculated by ssGSEA algorithm. Gene set for pericyte score calculation was described previously.^52,53^

### Statistical analyses

Two-tailed unpaired Student’s *t*-test and one-way ANOVA were used to determine significance. *P* value < 0.05 was considered statistically significant. Survival analysis was performed by Kaplan–Meier method, with the log-rank test for comparison. The cutoff point optimization of patients in TCGA and CGGA database was calculated by X-tile software. Univariate and multivariate analyses were performed using Cox-regression analyses by SPSS Statistics 23 (IBM, USA). All quantitative data are means ± SD.

## Supplementary information

Supplementary information, Fig. S1

Supplementary information, Fig. S2

Supplementary information, Fig. S3

Supplementary information, Fig. S4

Supplementary information, Fig. S5

Supplementary information, Fig. S6

Supplementary information, Fig. S7

Supplementary information, Fig. S8

Supplementary information, Table S1

Supplementary information, Table S2

Supplementary information, Table S3

Supplementary information, Table S4

Supplementary information, Table S5

Supplementary information, Table S6

Supplementary information, Table S7

Supplementary information, Table S8
