# Supplementary material for: Pericytes augment glioblastoma cell resistance to temozolomide through CCL5-CCR5 paracrine signaling
Source: Cell Res. 2021 Jul 8;31(10):1072–87. doi: 10.1038/s41422-021-00528-3 (PMC8486800; doi:10.1038/s41422-021-00528-3)
Supplement: Supplementary file 1 — Supplementary information, Fig. S1 [file 41422_2021_528_MOESM1_ESM.pdf]

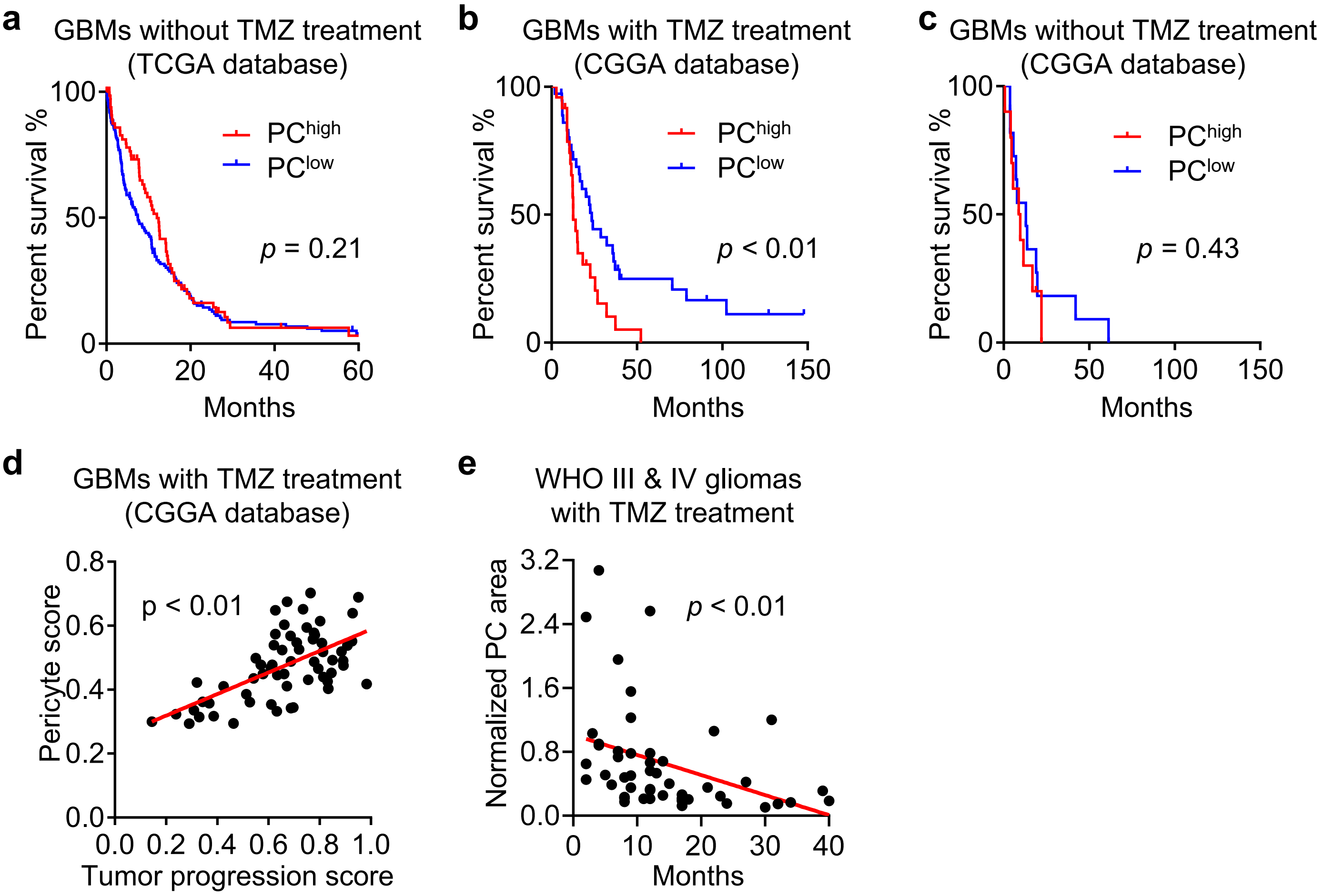

Fig. S1. Prognosis of GBM patients with or without TMZ treatment.

a Kaplan-Meier analysis of the overall survival (OS) of GBM patients without TMZ treatment stratified by pericyte (PC) score. Pericyte score was calculated according to the expression of pericyte markers in GBMs from the TCGA database (PC^{high}, n = 67, PC^{low}, n = 150). **b** Kaplan-Meier analysis of the overall survival (OS) of TMZ-treated GBM patients stratified by pericyte score. Pericyte score was calculated according to the expression of pericyte markers in GBMs from the CGGA database (PC^{high}, n = 24, PC^{low}, n = 37). **c** Kaplan-Meier analysis of the overall survival (OS) of GBM patients without TMZ treatment stratified by pericyte score (PC^{high}, n = 10, PC^{low}, n = 11). **d** Correlation analysis of pericyte score and tumor progression score in TMZ-treated GBMs from the CGGA database (n=61). Tumor progression score is constructed by ssGSEA model based on 14 signature genes derived from multi-focal GBM RNA sequencing. **e** Correlation analysis of normalized PC area and progression free survival (PFS) of WHO III & IV gliomas patients (n = 49) from Southwest Hospital.
