# Supplementary material for: Pericytes augment glioblastoma cell resistance to temozolomide through CCL5-CCR5 paracrine signaling
Source: Cell Res. 2021 Jul 8;31(10):1072–87. doi: 10.1038/s41422-021-00528-3 (PMC8486800; doi:10.1038/s41422-021-00528-3)
Supplement: Supplementary file 2 — Supplementary information, Fig. S2 [file 41422_2021_528_MOESM2_ESM.pdf]

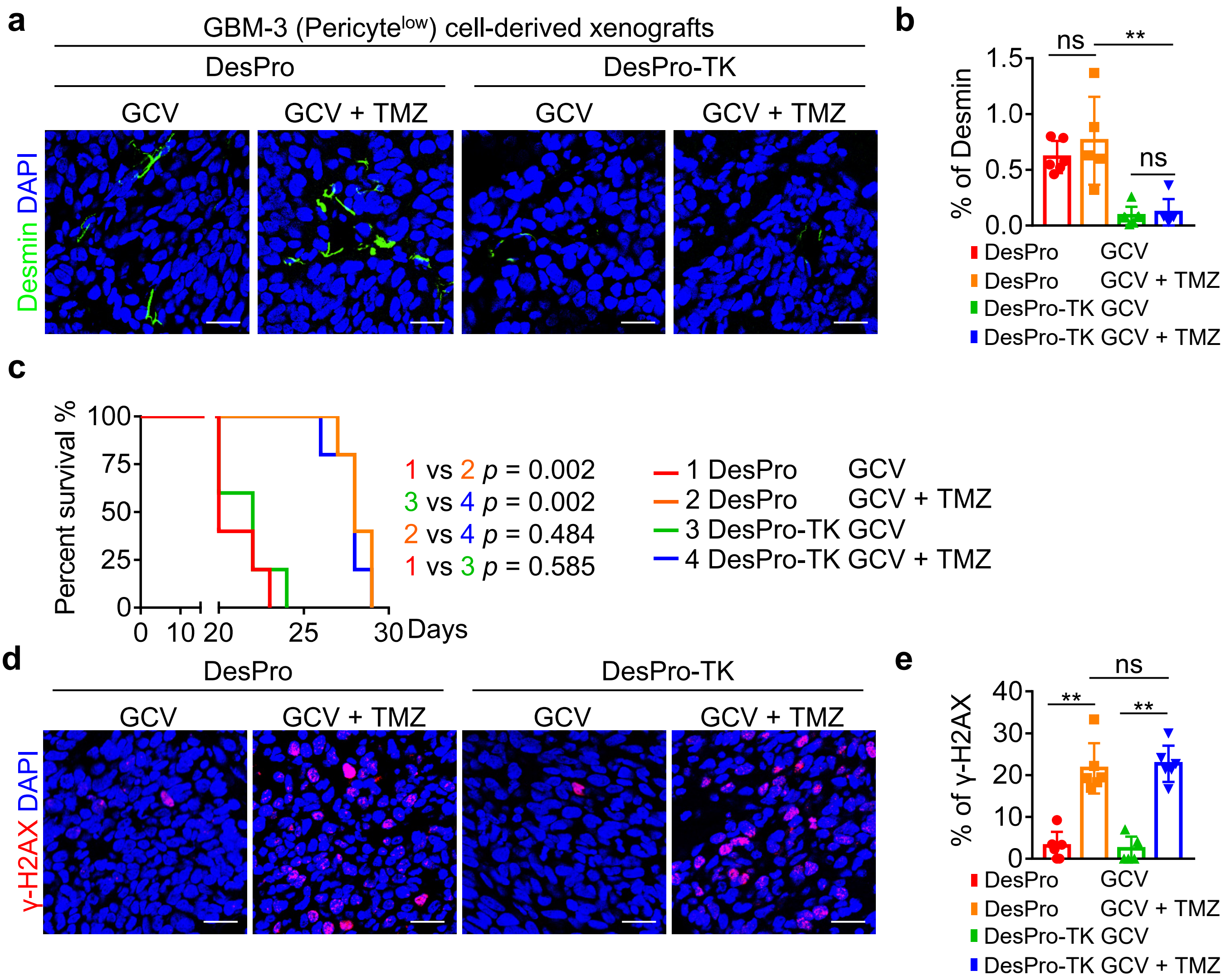

Fig. S2. Depletion of pericytes in pericyte^low GBMs showed minimal effect on the therapeutic efficacy of TMZ.

a, b Immunofluorescence staining (**a**) and quantification (**b**) of pericyte marker Desmin (green) in GBM-3 xenografts treated with GCV with or without TMZ. ns, not significant. $^{**}p < 0.01$. Scale bars, 25 μm. **c** Kaplan-Meier survival analysis of mice bearing GBM-3 (pericyte^low) xenografts with indicated treatment. n = 5 for each group. **d, e** Immunofluorescence staining (**d**) and quantification (**e**) of γ-H2AX (red) positive cells in GBM-3 xenografts treated with GCV with or without TMZ. ns, not significant. $^{**}p < 0.01$. Scale bars, 25 μm.
