# Supplementary material for: Pericytes augment glioblastoma cell resistance to temozolomide through CCL5-CCR5 paracrine signaling
Source: Cell Res. 2021 Jul 8;31(10):1072–87. doi: 10.1038/s41422-021-00528-3 (PMC8486800; doi:10.1038/s41422-021-00528-3)
Supplement: Supplementary file 3 — Supplementary information, Fig. S3 [file 41422_2021_528_MOESM3_ESM.pdf]

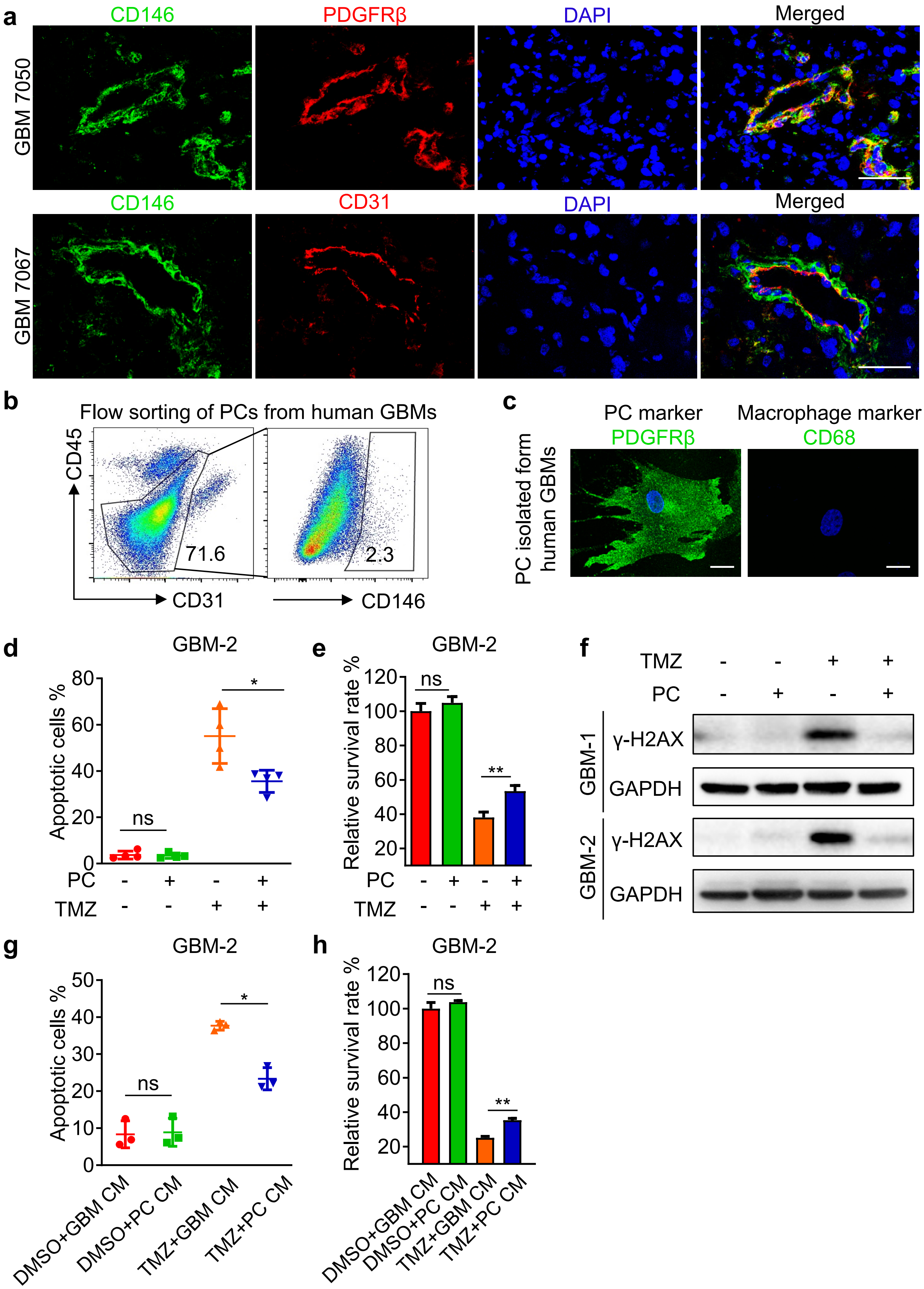

a
CD146
PDGFRβ
DAPI
Merged
GBM 7050
CD146
CD31
DAPI
Merged
GBM 7067
b
Flow sorting of PCs from human GBMs
CD45
CD31
71.6
2.3
CD146
c
PC marker
PDGFRβ
Macrophage marker
CD68
PC isolated form human GBMs
d
GBM-2
Apoptotic cells %
ns
*
PC - + - +
TMZ - - + +
e
GBM-2
Relative survival rate %
ns
**
PC - + - +
TMZ - - + +
f
TMZ - - + +
PC - + - +
GBM-1
γ-H2AX
GAPDH
GBM-2
γ-H2AX
GAPDH
g
GBM-2
Apoptotic cells %
ns
*
DMSO+GBM CM
DMSO+PC CM
TMZ+GBM CM
TMZ+PC CM
h
GBM-2
Relative survival rate %
ns
**
DMSO+GBM CM
DMSO+PC CM
TMZ+GBM CM
TMZ+PC CM

Fig. S3. Pericytes protect GBM cells from TMZ-induced apoptosis.

a Immunofluorescence staining of CD146 (green), PDGFRβ (red) and CD31 (red) in human GBMs. Scale bars, 50 μm. **b** Fluorescence-activated cell sorting (FACS) of $CD146^{+} CD31^{-} CD45^{-}$ pericytes from human GBMs. **c** Immunofluorescence staining of pericyte (PC) marker PDGFRβ and macrophage marker CD68 in $CD146^{+}CD31^{-}CD45^{-}$ cells sorted from human GBMs. Scale bars, 25 μm. **d, e** Apoptosis (**d**) and cell survival (**e**) of GBM-2 cells with or without pericyte co-culture and TMZ treatment. ns, not significant. $^{*}p < 0.05$, $^{**}p < 0.01$. **f** Immunoblot analysis of γ-H2AX and GAPDH in GBM-1 and GBM-2 cells with or without pericyte co-culture and TMZ treatment. **g, h** Apoptosis (**g**) and cell survival (**h**) of GBM-2 cells with or without pericyte CM stimulation and TMZ treatment. ns, not significant. $^{*}p < 0.05$, $^{**}p < 0.01$. Experiments (**c-h**) were performed independently for three times.
