# Supplementary material for: Pericytes augment glioblastoma cell resistance to temozolomide through CCL5-CCR5 paracrine signaling
Source: Cell Res. 2021 Jul 8;31(10):1072–87. doi: 10.1038/s41422-021-00528-3 (PMC8486800; doi:10.1038/s41422-021-00528-3)
Supplement: Supplementary file 4 — Supplementary information, Fig. S4 [file 41422_2021_528_MOESM4_ESM.pdf]

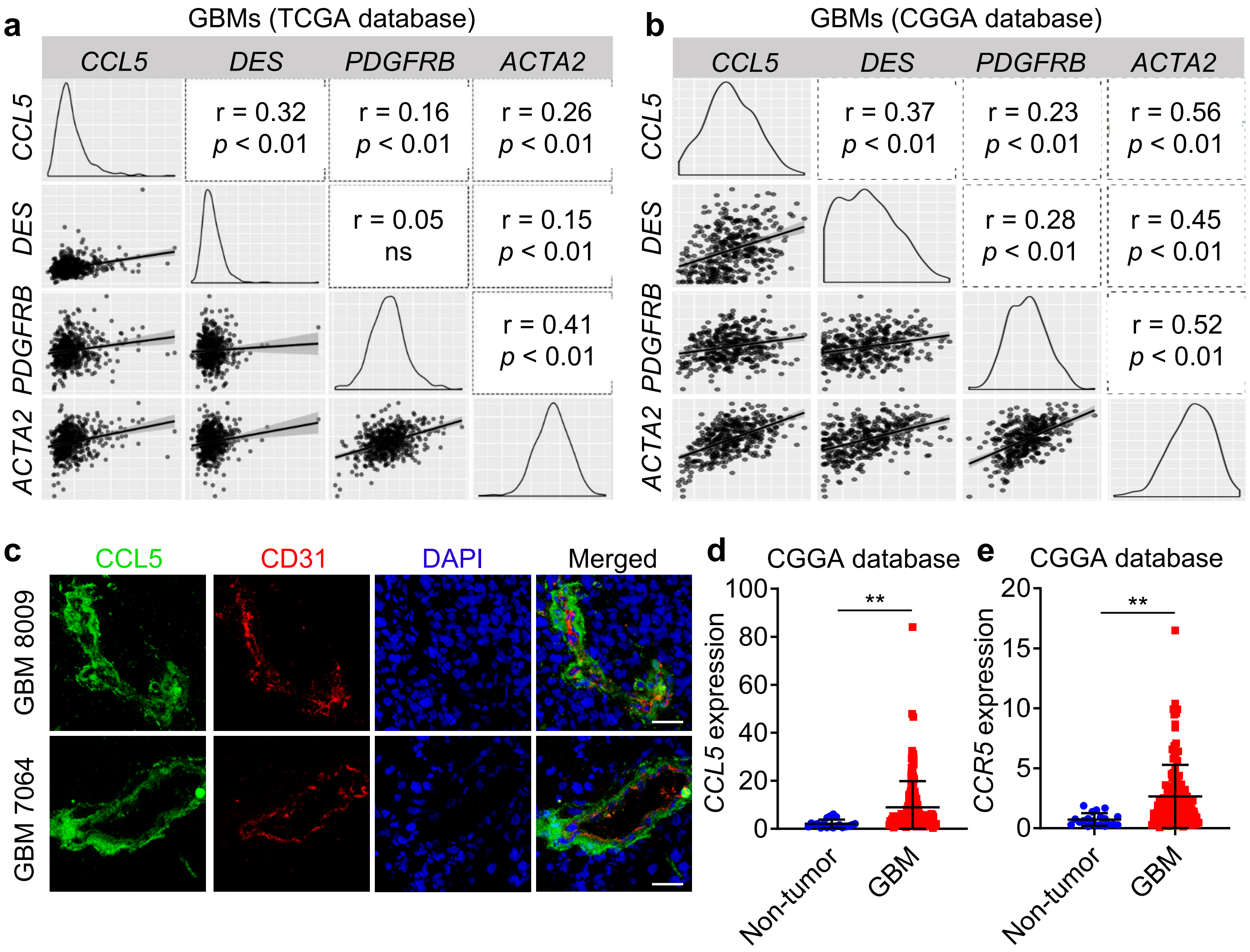

Fig. S4. Correlation of CCL5 and pericyte markers and the distribution of CCL5 in human GBMs.

a Bivariate correlation analysis showing positive correlations of *CCL5* and pericyte markers *DES, PDGFRB* and *ACTA2* expression in GBMs from the TCGA database (n = 538). ns, not significant. **b** Bivariate correlation analysis showing positive correlations of *CCL5* and pericyte markers *DES, PDGFRB* and *ACTA2* expression in GBMs from the CGGA database (n = 388). **c** Immunofluorescence staining of CCL5 (green) and CD31 (red) in human GBMs. Scale bars, 50 µm. **d, e** Expressions of *CCL5* (**d**) and *CCR5* (**e**) in human GBMs (n = 139) and non-tumor tissues (n = 20) from the CGGA database. **$p < 0.01$.
