# Supplementary material for: Pericytes augment glioblastoma cell resistance to temozolomide through CCL5-CCR5 paracrine signaling
Source: Cell Res. 2021 Jul 8;31(10):1072–87. doi: 10.1038/s41422-021-00528-3 (PMC8486800; doi:10.1038/s41422-021-00528-3)
Supplement: Supplementary file 5 — Supplementary information, Fig. S5 [file 41422_2021_528_MOESM5_ESM.pdf]

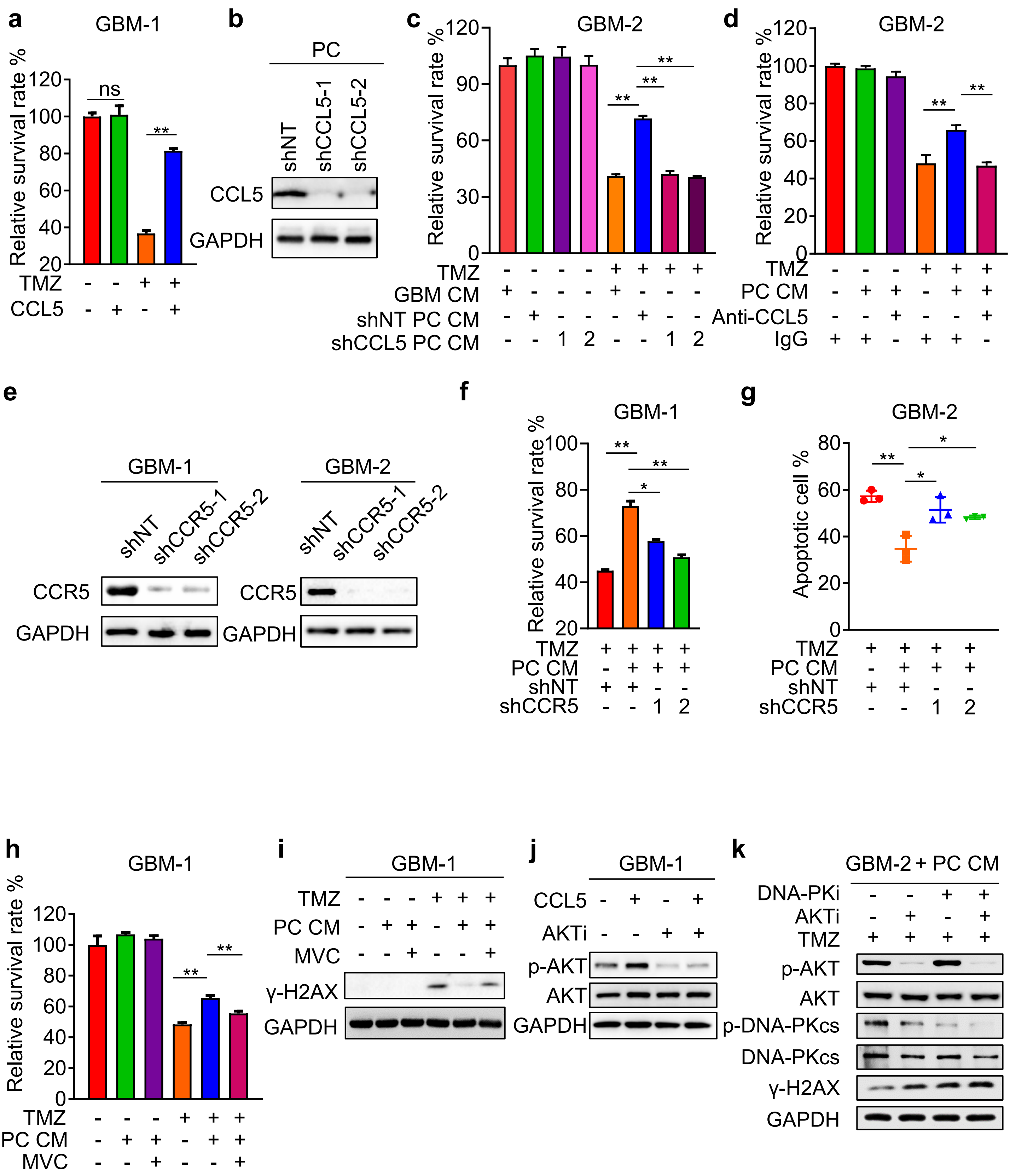

a
GBM-1
Relative survival rate %
120
100
80
60
40
20
ns
**
TMZ - - + +
CCL5 - + - +
b
PC
shNT
shCCL5-1
shCCL5-2
CCL5
GAPDH
c
GBM-2
Relative survival rate %
120
90
60
30
0
**
**
**
TMZ - - - - + + + +
GBM CM + - - - + - - -
shNT PC CM - + - - - + - -
shCCL5 PC CM - - 1 2 - - 1 2
d
GBM-2
Relative survival rate %
120
100
80
60
40
20
0
**
**
TMZ - - - + + +
PC CM - + + - + +
Anti-CCL5 - - + - - +
IgG + + - + + -
e
GBM-1
GBM-2
shNT
shCCR5-1
shCCR5-2
CCR5
GAPDH
f
GBM-1
Relative survival rate %
100
80
60
40
20
**
**
*
TMZ + + + +
PC CM - + + +
shNT + + - -
shCCR5 - - 1 2
g
GBM-2
Apoptotic cell %
80
60
40
20
0
**
*
*
TMZ + + + +
PC CM - + + +
shNT + + - -
shCCR5 - - 1 2
h
GBM-1
Relative survival rate %
120
100
80
60
40
20
0
**
**
TMZ - - - + + +
PC CM - + + - + +
MVC - - + - - +
i
GBM-1
TMZ - - - + + +
PC CM - + + - + +
MVC - - + - - +
γ-H2AX
GAPDH
j
GBM-1
CCL5 - + - +
AKTi - - + +
p-AKT
AKT
GAPDH
k
GBM-2 + PC CM
DNA-PKi - - + +
AKTi - + - +
TMZ + + + +
p-AKT
AKT
p-DNA-PKcs
DNA-PKcs
γ-H2AX
GAPDH

Fig. S5. Disruption of CCR5 signaling compromises the protective effect of pericytes on DNA damage induced by TMZ treatment.

a Cell survival analysis of GBM-1 cells with indicated treatments. GBM cells were stimulated with CCL5 (10 ng/ml) or control vehicle (PBS) followed by the administration of TMZ (500 μmol/L) or DMSO. ns, not significant. $^{**}p < 0.01$. **b** Immunoblot analysis of CCL5 expression in pericytes (PC) expressing shNT or shCCL5 (shCCL5-1 or shCCL5-2) lentiviral vectors. **c** Cell survival analysis of GBM-2 cells with indicated treatments. CM of pericytes expressing shNT or shCCL5 (sh-1 or sh-2) were collected and added to GBM cells. GBM cells were treated with TMZ (500 μmol/L) after pericyte CM addition. $^{**}p < 0.01$. **d** Cell survival analysis of GBM-2 cells with indicated treatments. Pericyte CM was pretreated with neutralizing anti-CCL5 antibody or IgG before added to GBM cells. GBM cells were treated with TMZ (500 μmol/L) after pericyte CM addition. $^{**}p < 0.01$. **e** Immunoblot analysis of CCR5 expression in GBM-1 and GBM-2 cells expressing shNT or shCCR5 (shCCR5-1 or shCCR5-2) lentiviral vectors. **f** Cell survival analysis of GBM-1 cells with indicated treatments. GBM cells expressing shNT or shCCR5 (sh-1 or sh-2) were pretreated with pericyte CM or control medium (GBM cell CM) followed by TMZ (500 μmol/L) treatment. $^{*}p < 0.05$; $^{**}p < 0.01$. **g** Apoptosis analysis of GBM-2 cells with indicated treatments. GBM cells expressing shNT or shCCR5 (sh-1 or sh-2) were pretreated with pericyte CM or control medium followed by TMZ (500 μmol/L) treatment. $^{*}p < 0.05$; $^{**}p < 0.01$. **h** Cell survival analysis of GBM-1 cells with indicated treatments. GBM-1 cells were pretreated with MVC (500 nmol/L) or DMSO for 1 hour and followed by pericyte CM stimulation. Apoptosis and cell survival analyses were performed 48 hours after TMZ treatment. $^{**}p < 0.01$. **i** Immunoblot analysis of γ-H2AX and GAPDH in GBM-1 cells with indicated treatments. **j** Immunoblot analysis of phosphorylated-AKT (Ser473) and AKT in GBM-1 cells pretreated with or without CCL5 (10 ng/ml) followed by the treatment of AKTi (AKT inhibitor) MK-2206 (500 nmol/L). **k** Immunoblot analysis of phosphorylated-AKT (Ser473), AKT, phosphorylated-DNA-PKcs (Ser2056), DNA-PKcs, γ-H2AX in GBM-2 cells with indicated treatments. DNA-PKi (DNA-PKcs inhibitor) represents KU-57788, AKTi (AKT inhibitor) represents MK-2206.
