# Supplementary material for: Pericytes augment glioblastoma cell resistance to temozolomide through CCL5-CCR5 paracrine signaling
Source: Cell Res. 2021 Jul 8;31(10):1072–87. doi: 10.1038/s41422-021-00528-3 (PMC8486800; doi:10.1038/s41422-021-00528-3)
Supplement: Supplementary file 6 — Supplementary information, Fig. S6 [file 41422_2021_528_MOESM6_ESM.pdf]

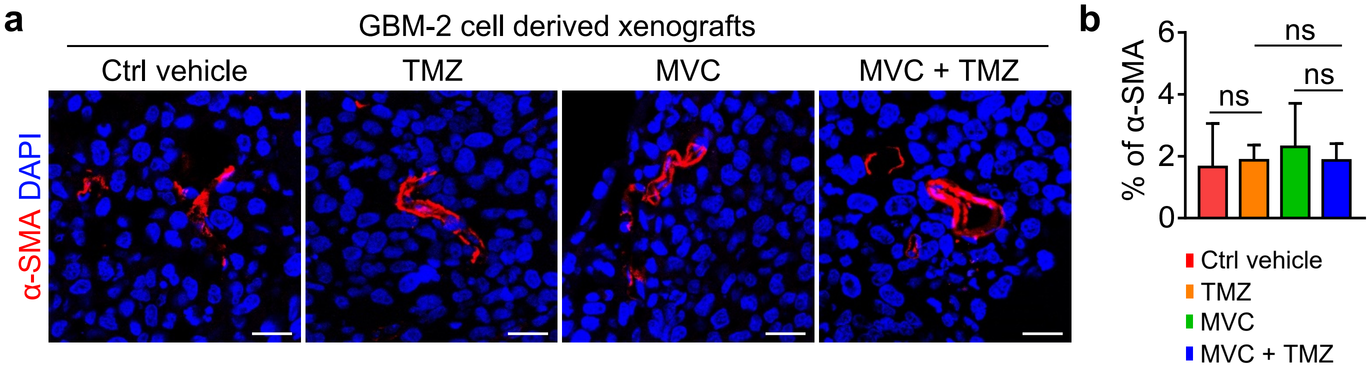

Fig. S6. Treatment of MVC and TMZ has no effect on pericyte area.

a, b Immunofluorescence staining (**a**) and quantification (**b**) of pericyte marker α-SMA (red) in GBM-2 xenografts treated with MVC with or without TMZ. ns, not significant. Scale bars, 25 µm.
