# Supplementary material for: Pericytes augment glioblastoma cell resistance to temozolomide through CCL5-CCR5 paracrine signaling
Source: Cell Res. 2021 Jul 8;31(10):1072–87. doi: 10.1038/s41422-021-00528-3 (PMC8486800; doi:10.1038/s41422-021-00528-3)
Supplement: Supplementary file 7 — Supplementary information, Fig. S7 [file 41422_2021_528_MOESM7_ESM.pdf]

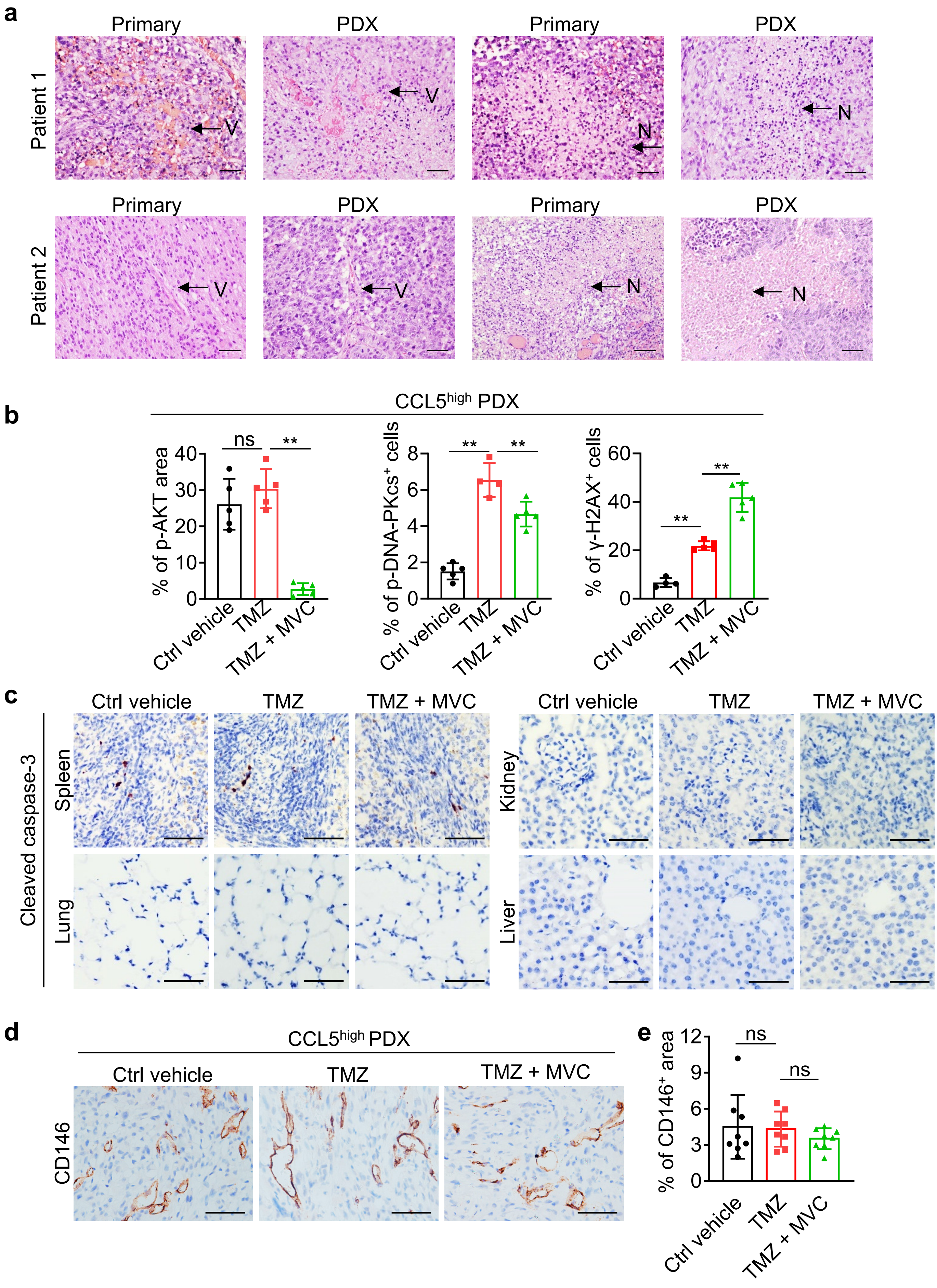
a
Primary
PDX
Primary
PDX
Patient 1
V
V
N
N
Primary
PDX
Primary
PDX
Patient 2
V
V
N
N
b
CCL5high PDX
% of p-AKT area
40
30
20
10
0
ns
**
Ctrl vehicle
TMZ
TMZ + MVC
% of p-DNA-PKcs+ cells
8
6
4
2
0
**
**
Ctrl vehicle
TMZ
TMZ + MVC
% of γ-H2AX+ cells
60
40
20
0
**
**
Ctrl vehicle
TMZ
TMZ + MVC
c
Ctrl vehicle
TMZ
TMZ + MVC
Ctrl vehicle
TMZ
TMZ + MVC
Cleaved caspase-3
Spleen
Lung
Kidney
Liver
d
CCL5high PDX
Ctrl vehicle
TMZ
TMZ + MVC
CD146
e
% of CD146+ area
12
9
6
3
0
ns
ns
Ctrl vehicle
TMZ
TMZ + MVC

Fig. S7. Adjuvant treatment of MVC with TMZ impairs DNA damage repair in GBM PDXs and displays negligible gross toxicity.

a H&E staining of primary GBMs (left panel) and the corresponding PDXs (right panel). Representative morphologies of vessels (V) and necrosis (N) were indicated by arrows. Scale bars, 100 μm. **b** Quantification of phosphorylated-AKT (Ser473), phosphorylated-DNA-PKcs (Ser2056) and γ-H2AX (Ser139) in $CCL5^{high}$ PDXs treated with TMZ with or without MVC as an adjuvant agent. ns, not significant, $^{**}p < 0.01$. **c** Immunohistochemistry staining of cleaved caspase-3 in organs with CCR5 expression (spleens and lungs) and in organs participated in drug metabolism (livers and kidneys) in mice treated with TMZ with or without MVC. n = 5 for each group. Scale bars, 100 μm. **d, e** Representative images of immunohistochemistry staining (**d**) and quantification (**e**) of pericyte marker CD146 in $CCL5^{high}$ PDXs treated with TMZ with or without MVC. ns, not significant. Scale bars, 100 μm.
