# Supplementary material for: Pericytes augment glioblastoma cell resistance to temozolomide through CCL5-CCR5 paracrine signaling
Source: Cell Res. 2021 Jul 8;31(10):1072–87. doi: 10.1038/s41422-021-00528-3 (PMC8486800; doi:10.1038/s41422-021-00528-3)
Supplement: Supplementary file 8 — Supplementary information, Fig. S8 [file 41422_2021_528_MOESM8_ESM.pdf]

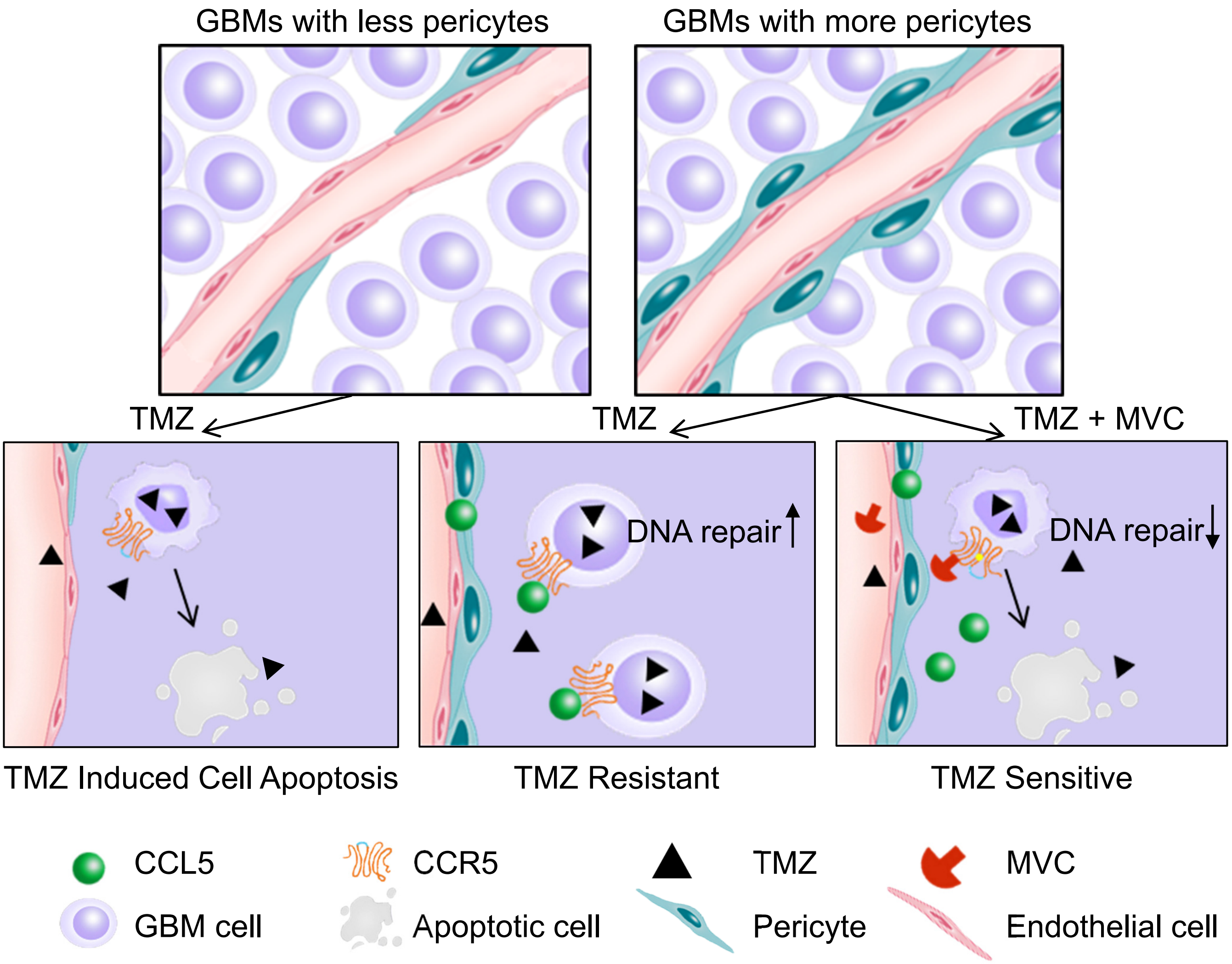

Fig. S8. Personalized treatment of MVC as a chemo-sensitizing agent in GBMs.

Pericytes act as an environmental stimulator to facilitate DNA repair. In GBMs with sparse pericytes, TMZ treatment alone induces cytotoxicity and apoptosis of GBM cells. In GBMs enriched with pericytes, pericyte-secreted CCL5 acts on CCR5 expressed on GBM cells to enhance DNA repair and induces chemoresistance of TMZ. Personalized treatment of CCR5 antagonist MVC effectively compromises CCL5-CCR5 signaling activation and impairs pericyte protection on survival of GBM cells. Therefore, administration of MVC as chemo-sensitizing agent reverses TMZ resistance in pericyte-high GBMs.
