# Supplementary material for: Pericytes augment glioblastoma cell resistance to temozolomide through CCL5-CCR5 paracrine signaling
Source: Cell Res. 2021 Jul 8;31(10):1072–87. doi: 10.1038/s41422-021-00528-3 (PMC8486800; doi:10.1038/s41422-021-00528-3)
Supplement: Supplementary file 9 — Supplementary information, Table S1 [file 41422_2021_528_MOESM9_ESM.pdf]

Table S1. ELISA of CD146 recombinant protein using CD146 antibody.

Dilution ratio	OD value (450 nm)		
	His-CD146	Fc-CD146	His-Control
Empty	0.046	0.050	0.051
1:1000	2.256	2.198	0.060
1:2000	2.218	1.985	0.059
1:4000	1.479	1.269	0.055
1:8000	0.802	0.622	0.056
1:16000	0.302	0.156	0.052
1:32000	0.148	0.085	0.053
1:64000	0.095	0.063	0.051

Abbreviations: ELISA, enzyme linked immunosorbent assay; OD, optical density; Fc, Fc region of human IgG1; His, polyhistidine tag.
