# Supplementary material for: Pericytes augment glioblastoma cell resistance to temozolomide through CCL5-CCR5 paracrine signaling
Source: Cell Res. 2021 Jul 8;31(10):1072–87. doi: 10.1038/s41422-021-00528-3 (PMC8486800; doi:10.1038/s41422-021-00528-3)
Supplement: Supplementary file 10 — Supplementary information, Table S2 [file 41422_2021_528_MOESM10_ESM.pdf]

Table S2. Upregulated cytokines in pericytes relative to control GBM cells.

Cytokines	Average mRNA level							
	PC-1	PC-2	PC-3	PC-4	GBM-1	GBM-2	GBM-4	GBM-5
CCL5	7.02	7.53	7.94	10.54	NA.	2.39	NA.	-2.97
CCL20	7.94	10.39	7.27	9.68	-0.43	-1.51	2.87	2.78
EBI3	7.22	7.53	6.65	8.47	3.64	1.81	2.91	1.14
IL10	2.41	1.48	5.45	-0.77	NA.	-3.29	0.84	-2.97
TNFSF15	4.84	4.59	3.46	1.15	NA.	-7.68	-8.07	-2.97
CCL4	4.01	0.52	3.89	8.98	NA.	0.57	-2.94	-2.97
AREG	1.80	4.45	4.98	4.99	-0.69	-0.39	2.19	5.40
IL18	1.25	0.99	-0.76	3.18	-0.69	0.12	-4.75	6.16
IL1B	NA.	-2.16	12.78	6.29	6.89	4.98	2.71	7.07
CXCL9	-3.26	-1.51	NA.	-5.18	-0.75	2.55	-1.71	4.02
TNF	1.55	-1.65	-1.40	0.95	-0.69	-0.86	NA.	1.26
CCL14	0.87	-1.93	-0.60	-1.48	-0.69	NA.	NA.	NA.
CCL18	NA.	NA.	NA.	NA.	NA.	NA.	NA.	NA.
IL1A	NA.	NA.	NA.	NA.	NA.	NA.	NA.	NA.
CCL8	-3.26	1.37	NA.	0.77	-2.24	NA.	NA.	-2.32
CCL3	-2.33	-1.38	-1.43	-1.40	0.27	NA.	-2.00	-2.75
CXCL16	-0.87	-0.28	-1.43	-3.49	1.03	-0.41	-0.12	-0.56
OSM	-2.56	-1.27	-0.41	-4.18	NA.	-0.68	NA.	NA.

Abbreviations: PC, pericyte; GBM, glioblastoma; NA., not available.
