# Supplementary material for: Pericytes augment glioblastoma cell resistance to temozolomide through CCL5-CCR5 paracrine signaling
Source: Cell Res. 2021 Jul 8;31(10):1072–87. doi: 10.1038/s41422-021-00528-3 (PMC8486800; doi:10.1038/s41422-021-00528-3)
Supplement: Supplementary file 11 — Supplementary information, Table S3 [file 41422_2021_528_MOESM11_ESM.pdf]

Table S3. Cox-regression analyses of the overall survival of GBM patients from the TCGA database.

Factor	Univariate		Multivariate	
	HR (95% CI)	*p* value	HR (95% CI)	*p* value
Age (Years)	1.790 (1.477-2.170)	0.00	1.836 (1.511-2.232)	0.00
Sex	0.909 (0.755-1.094)	0.31	0.860 (0.712-1.039)	0.12
IDH1 mutation status	0.674 (0.568-0.801)	0.00	0.727 (0.604-0.876)	0.00
TMZ	0.588 (0.486-0.712)	0.00	0.577 (0.469-0.709)	0.00
CCL5	1.122 (1.011-1.244)	0.03	1.133 (1.017-1.261)	0.02

Abbreviations: GBM, glioblastoma; HR, harzard ratio; CI, confidence interval; IDH1, isocitrate dehydrogenase type 1; TMZ, temozolomide; CCL5, C-C motif chemokine ligand 5.
