# Supplementary material for: Pericytes augment glioblastoma cell resistance to temozolomide through CCL5-CCR5 paracrine signaling
Source: Cell Res. 2021 Jul 8;31(10):1072–87. doi: 10.1038/s41422-021-00528-3 (PMC8486800; doi:10.1038/s41422-021-00528-3)
Supplement: Supplementary file 12 — Supplementary information, Table S4 [file 41422_2021_528_MOESM12_ESM.pdf]

Table S4. Serological parameters of livers and kidneys in mice treated with TMZ with or without MVC.

	Vehicle	MVC	TMZ	MVC + TMZ	Normal range
Urea (mmol/L)	6.78 ± 0.43	7.00 ± 1.3	7.28 ± 0.60	7.42 ± 0.64	4.3-10.7
ALT (IU/L)	40.34 ± 5.02	38.54 ± 6.96	32.68 ± 3.58	33.16 ± 3.71	24-77
AST (IU/L)	153.38 ± 40.16	142.54 ± 12.42	116.92 ± 11.47	105.32 ± 6.85	53-269
ALP (IU/L)	94.80 ± 27.48	89.40 ± 14.77	72.20 ± 5.36	118.00 ± 9.3	45-199
TP (g/L)	59.16 ± 3.94	56.96 ± 2.13	62.28 ± 2.54	57.34 ± 2.39	40-60
ALB (g/L)	25.24 ± 1.88	26.08 ± 1.84	27.96 ± 1.69	27.82 ± 0.93	25-48
TBIL (μmol/L)	1.80 ± 0.76	1.38 ± 0.47	1.90 ± 0.38	1.36 ± 0.54	1.71-15.39

Abbreviations: MVC, maraviroc; TMZ, temozolomide; IU, international unit; ALT, alanine aminotransferase; AST, aspartate aminotransferase; ALP, alkaline phosphatase; TP, total protein; ALB, albumin; TBIL, total bilirubin.
