# Supplementary material for: Pericytes augment glioblastoma cell resistance to temozolomide through CCL5-CCR5 paracrine signaling
Source: Cell Res. 2021 Jul 8;31(10):1072–87. doi: 10.1038/s41422-021-00528-3 (PMC8486800; doi:10.1038/s41422-021-00528-3)
Supplement: Supplementary file 13 — Supplementary information, Table S5 [file 41422_2021_528_MOESM13_ESM.pdf]

Table S5. Clinicopathological information of primary GBM cells used in this study.

GBM cells	Grade	IDH1 mutation status	MGMT promoter	Molecular subtypes
GBM-1	WHO IV	WT	Unmethylated	Mesenchymal
GBM-2 (pericyte^{high})	WHO IV	WT	Unmethylated	Mesenchymal
GBM-3 (Derived from pericyte^{low} PDXs)	WHO IV	WT	Unmethylated	Proneural

Abbreviations: GBM, glioblastoma; IDH1, isocitrate dehydrogenase type 1; MGMT, O^6-methylguanine-DNA methyltransferase; WT, wide type; PDXs, patient-derived xenografts.
