# Supplementary material for: Pericytes augment glioblastoma cell resistance to temozolomide through CCL5-CCR5 paracrine signaling
Source: Cell Res. 2021 Jul 8;31(10):1072–87. doi: 10.1038/s41422-021-00528-3 (PMC8486800; doi:10.1038/s41422-021-00528-3)
Supplement: Supplementary file 14 — Supplementary information, Table S6 [file 41422_2021_528_MOESM14_ESM.pdf]

Table S6. Short hairpin RNA sequences used for lentiviral vector construction.

Vector	Sequence (5' to 3')
shNT	TTCTCCGAACGTGTCACGT
shCCL5-1	GAAATGGGTTCGGGAGTACAT
shCCL5-2	GTATTTCTACACCAGTGGCAA

Vector	Sequence (5' to 3')
shNT	TTCTCCGAACGTGTCACGT
shCCR5-1	CGGGAATCCTAAAAACTCT
shCCR5-2	GAGCGAGCAAGCTCAGTTT

Abbreviations: shNT, non-targeting control shRNA.
