# Supplementary material for: Pericytes augment glioblastoma cell resistance to temozolomide through CCL5-CCR5 paracrine signaling
Source: Cell Res. 2021 Jul 8;31(10):1072–87. doi: 10.1038/s41422-021-00528-3 (PMC8486800; doi:10.1038/s41422-021-00528-3)
Supplement: Supplementary file 15 — Supplementary information, Table S7 [file 41422_2021_528_MOESM15_ESM.pdf]

Table S7. Primers used for qRT-PCR analyses in this study.

Gene	Forward primers (5’ to 3’)	Reverse primers (5’ to 3’)
CCL5	CCTGCTGCTTTGCCTACATTGC	ACACACTTGGCGGTTCTTTCGG
CCL20	AAGTTGTCTGTGTGCGCAAATCC	CCATTCCAGAAAAGCCACAGTTTT
EBI3	CTGGATCCGTTACAAGCGTCAG	CACTTGGACGTAGTACCTGGCT
IL10	TCTCCGAGATGCCTTCAGCAGA	TCAGACAAGGCTTGGCAACCCA
TNFSF15	CACCACATACCTGCTTGTCAGC	TCTCCGTCTGCTCTAAGAGGTG
CCL4	GCTTCCTCGCAACTTTGTGGTAG	GGTCATACACGTACTCCTGGAC
AREG	GCACCTGGAAGCAGTAACATGC	GGCAGCTATGGCTGCTAATGCA
IL18	GATAGCCAGCCTAGAGGTATGG	CCTTGATGTTATCAGGAGGATTCA
IL1B	CCACAGACCTTCCAGGAGAATG	GTGCAGTTCAGTGATCGTACAGG
CXCL9	CTGTTCCTGCATCAGCACCAAC	TGAACTCCATTCTTCAGTGTAGCA
TNF	CTCTTCTGCCTGCTGCACTTTG	ATGGGCTACAGGCTTGTCACTC
CCL14	TGATGTCAAAGCTTCCACTGGAAA	GAGTGAACACGGGATGCTTTGTG
CCL18	GTTGACTATTCTGAAACCAGCCC	GTCGCTGATGTATTTCTGGACCC
IL1A	TGTATGTGACTGCCCAAGATGAAG	AGAGGAGGTTGGTCTCACTACC
CCL8	TATCCAGAGGCTGGAGAGCTAC	TGGAATCCCTGACCCATCTCTC
CCL3	ACTTTGAGACGAGCAGCCAGTG	TTTCTGGACCCACTCCTCACTG
CXCL16	CCTATGTGCTGTGCAAGAGGAG	CTGGGCAACATAGAGTCCGTCT
OSM	GAAAGAGTACCGCGTGCTCCTT	CTCTCAGTTTAGGAACATCCAGG

Abbreviations: qRT-PCR, quantitative real-time PCR.
