# Supplementary material for: Pericytes augment glioblastoma cell resistance to temozolomide through CCL5-CCR5 paracrine signaling
Source: Cell Res. 2021 Jul 8;31(10):1072–87. doi: 10.1038/s41422-021-00528-3 (PMC8486800; doi:10.1038/s41422-021-00528-3)
Supplement: Supplementary file 16 — Supplementary information, Table S8 [file 41422_2021_528_MOESM16_ESM.pdf]

Table S8. Clinicopathological information of GBM PDXs used in this study.

PDXs	Grade	Sex	Age (Years)	IDH1 mutation status	MGMT promoter	Molecular subtypes
$CCL5^{high}$	WHO IV	Male	37	WT	Unmethylated	Mesenchymal
$CCL5^{low}$	WHO IV	Male	46	WT	Unmethylated	Proneural

Abbreviations: GBM, glioblastoma; PDXs, patient-derived xenografts; IDH1, isocitrate dehydrogenase type 1; MGMT, O^6-methylguanine-DNA methyltransferase; WT, wide type.
